# Real-time imaging of single neuronal cell apoptosis in patients with glaucoma

**DOI:** 10.1093/brain/awx088

**Published:** 2017-04-26

**Authors:** Maria F. Cordeiro, Eduardo M. Normando, M. Jorge Cardoso, Serge Miodragovic, Seham Jeylani, Benjamin M. Davis, Li Guo, Sebastien Ourselin, Roger A’Hern, Philip A. Bloom

**Affiliations:** 1 Glaucoma and Retinal Neurodegeneration Group, Department of Visual Neuroscience, UCL Institute of Ophthalmology, London EC1V 9EL, UK; 2 The Western Eye Hospital, Imperial College Healthcare NHS Trust (ICHNT), London NW1 5QH, UK; 3 The Imperial College Ophthalmic Research Group (ICORG), Imperial College London NW1 5QH, UK; 4 Translational Imaging Group, Centre for Medical Image Computing, University College London, Wolfson House, Stephenson Way, London, NW1 2HE London, UK; 5 81 Hillier Road, London SW11 6AX, UK

**Keywords:** apoptosis, retinal imaging, glaucoma, real-time visualization

## Abstract

**See Herms and Schön (doi:10.1093/brain/awx100) for a scientific commentary on this article.**

Retinal cell apoptosis occurs in many ocular neurodegenerative conditions including glaucoma—the major cause of irreversible blindness worldwide. Using a new imaging technique that we have called DARC (detection of apoptosing retinal cells), which until now has only been demonstrated in animal models, we assessed if annexin 5 labelled with fluorescent dye DY-776 (ANX776) could be used safely in humans to identify retinal cell apoptosis. Eight patients with glaucomatous neurodegeneration and evidence of progressive disease, and eight healthy subjects were randomly assigned to intravenous ANX776 doses of 0.1, 0.2, 0.4 and 0.5 mg in an open-label, phase 1 clinical trial. In addition to assessing the safety, tolerability and pharmacokinetics of ANX776, the study aimed to explore whether DARC could successfully visualize individual retinal cell apoptosis *in vivo* in humans, with the DARC count defined as the total number of unique ANX776-labelled spots. DARC enabled retinal cell apoptosis to be identified in the human retina using ANX776. Single ANX776-labelled cells were visualized in a dose-dependent pattern (*P < *0.001) up to 6 h after injection. The DARC count was significantly higher (2.37-fold, 95% confidence interval: 1.4–4.03, *P = *0.003) in glaucoma patients compared to healthy controls, and was significantly (*P = *0.045) greater in patients who later showed increasing rates of disease progression, based on either optic disc, retinal nerve fibre layer or visual field parameters. Additionally, the DARC count significantly correlated with decreased central corneal thickness (Spearman’s R = −0.68, *P = *0.006) and increased cup-disc ratios (Spearman’s R = 0.47, *P = *0.038) in glaucoma patients and with increased age (Spearman’s R = 0.77, *P = *0.001) in healthy controls. Finally, ANX776 was found to be safe and well-tolerated with no serious adverse events, and a short half-life (10–36 min). This proof-of-concept study demonstrates that retinal cell apoptosis can be identified in the human retina with increased levels of activity in glaucomatous neurodegenerative disease. To our knowledge, this is the first time individual neuronal apoptosis has been visualized *in vivo* in humans and is the first demonstration of detection of individual apoptotic cells in a neurodegenerative disease. Furthermore, our results suggest the level of apoptosis (‘DARC count’) is predictive of disease activity, indicating the potential of DARC as a surrogate marker. Although further trials are clearly needed, this study validates experimental findings supporting the use of DARC as a method of detection and monitoring of patients with glaucomatous neurodegeneration, where retinal ganglion cell apoptosis is an established process and where there is a real need for tools to non-invasively assess treatment efficacy.

## Teaser

Glaucoma is often diagnosed late when vision loss has already occurred. Cordeiro *et al*. report a new fluorescent marker for retinal imaging that can safely visualise real-time *in vivo* neuronal apoptosis in patients. Increased labelling is observed in patients with progressive neurodegenerative disease compared to healthy controls.

## Introduction

Glaucoma is the leading cause of irreversible blindness worldwide attributed to the death of retinal nerve cells, specifically, retinal ganglion cells (RGC) (Cordeiro *et al.*, 2004; Tham *et al.*, 2014). It is considered a multifactorial neurodegenerative disorder of which the exact pathophysiology is unclear, but is characterized as a chronic progressive optic neuropathy associated with cupping of the optic-nerve head and classically, loss of peripheral vision (Kwon *et al.*, 2009). It is an age-dependent disease that has an increasing worldwide prevalence estimated at 3.54% in the over-40s (Tham *et al.*, 2014) and affects over 60 million people of whom more than 10% are bilaterally blind (Kyari *et al.*, 2013). The high rate of sight loss is due to the late presentation of patients with glaucoma as the disease is asymptomatic, and is often diagnosed late when significant vision loss has already occurred (Varma *et al.*, 2011).

One potential process that has been highlighted as an early marker of glaucomatous disease, is RGC apoptosis (Garcia-Valenzuela *et al.*, 1995; Quigley *et al.*, 1995; Kerrigan *et al.*, 1997; Kerrigan-Baumrind *et al.*, 2000; Cordeiro *et al.*, 2004; Quigley, 2011). Until now, it has not been possible to assess individual neuronal cell apoptosis in patients, despite advances in its detection.

Annexin 5 (ANX, encoded by *ANXA5*) is an endogenous 36 kDa protein ubiquitously expressed in humans, which in the presence of calcium, has a high affinity for phosphatidylserine exposed on apoptotic cell membranes. ANX is used in *in vitro* assays to measure apoptosis, and when radiolabelled has been used to identify apoptosis clinically, but not at single cell resolution (Vangestel *et al.*, 2011; Smith and Smith, 2012). The eye, compared to any other organ in the body, provides a distinct and unique opportunity to directly observe microscopic processes through clear optical media. Furthermore, due to the non-invasive nature of retinal imaging, these processes may be imaged repeatedly and longitudinally over time (Cordeiro *et al.*, 2004). Fluorescence imaging is routinely used in ophthalmology for the assessment of retinal disorders using the intravenous dyes fluorescein sodium and indocyanine green for angiography (Keane and Sadda, 2010).

Our group has investigated the real-time *in vivo* identification of retinal cell apoptosis using fluorescently labelled ANX and a technique we have termed DARC (detection of apoptosing retinal cells) (Cordeiro *et al.*, 2004). Using DARC, we have successfully visualized individual RGC apoptosis in different experimental models of neurodegeneration including Alzheimer’s and Parkinson’s disease, optic neuropathy, retinal neurodegeneration and glaucoma, previously shown only at post-mortem (Kerrigan *et al.*, 1997; Okisaka *et al.*, 1997), and demonstrated its ability to test treatment efficacy (Cordeiro *et al.*, 2004, 2010; Guo *et al.*, 2005, 2006, 2007, 2014; Maass *et al.*, 2007; Schmitz-Valckenberg *et al.*, 2008, 2010; Galvao *et al.*, 2014; Salt *et al.*, 2014; Normando *et al.*, 2016). Based on the collective preclinical evidence of DARC effectivity in assessing disease activity and therapeutic response (Supplementary Table 1), this technology has now been taken forward to the clinic, to investigate its potential application in humans.

Here, we report the results of a proof-of-concept study that sets out to explore (i) whether DARC could successfully identify individual retinal cell apoptosis in humans; and (ii) whether there was a difference in activity between healthy and glaucomatous eyes, while simultaneously establishing safety, tolerability and pharmacokinetics of intravenously administered fluorescently-labelled annexin 5 (ANX776).

## Materials and methods

### Study participants

The trial was conducted at The Western Eye Hospital, Imperial College Healthcare NHS Trust, as a single-centre, open-label study with subjects each receiving a single intravenous injection of ANX776. Both healthy and progressing glaucoma subjects were recruited to the trial (ClinicalTrials.gov number NCT02394613), with informed consent being obtained according to the Declaration of Helsinki after the study was approved by the Brent Research Ethics Committee. Healthy subjects were recruited through hospital advertisements. Enrolment was performed once sequential participants were considered eligible, according to the inclusion and exclusion criteria detailed in Supplementary Table 2. Briefly, healthy subjects were included if: there was no ocular or systemic disease, as confirmed by their GP; there was no evidence of any glaucomatous process either with optic disc, retinal nerve fibre layer (RNFL) or visual field abnormalities and with normal intraocular pressure; and they had repeatable and reliable imaging and visual fields. All glaucoma subjects were already under the care of the glaucoma department at the Western Eye Hospital, and were considered for inclusion in the study if they were found to have no ocular or systemic disease other than glaucoma with a minimum of three recent, sequential assessments with optic disc tomography (Heidelberg Retina Tomograph III), retinal optical coherence tomography (OCT) (Spectralis SD OCT, software version 6.0.0.2; Heidelberg Engineering) and standard automated perimetry (HFA 640i, Humphrey Field Analyzer; Carl Zeiss Meditec) using the Swedish interactive threshold algorithm standard 24-2. Eligible glaucoma subjects were required to show evidence of progressive disease in at least one eye of any parameter summarized in Table 1. OCT parameters included RNFL measurements at three different diameters from the optic disc (3.5, 4.1 and 4.7 mm) and Bruch’s membrane opening minimum rim width, and used in-built instrument software to compute glaucoma progression. Where this was not possible due to the duration of the pre-intervention period of assessment as in the case of Heidelberg retinal tomograph rim area, visual field mean deviation and visual field index, ordinary least squares regression was used to calculate linear rates of change of each parameter with time (Pathak *et al.*, 2013; Wang *et al.*, 2013). Glaucoma progression was defined by a significant (^*^P < 0.05; ^**^P < 0.01) negative slope in the rate of progression of each parameter identified in Table 1 and Supplementary Table 8.

**Table 1:** Glaucoma progression parameters

Glaucoma baseline rate of progression parameters (*P* < 0.05)								
Subject	Eye	HRT	OCT				SAP	
		HRT rim area mm^2^/year	RNFL 3.5 μm/year	RNFL 4.1 μm/year	RNFL 4.7 μm/year	MRW μm/year	MD dB/year	VFI %/year
5	R	+	+					
	L		+					
6	R	+	+					
	L							
7	R		+				+	
	L						+	
8	R		+	+	+	+		
	L		+					
9	R			+		+		
	L							
11	L		+	+				
13	R					+		
	L							
14	R		+	+				
	L							

Glaucoma progression parameters: In-built instrument software was used to compute rates of progression with OCT using RNFL measurements at three different diameters from the optic disc (3.5, 4.1 and 4.7 mm), and Bruch’s membrane opening minimum rim width (MRW). For Heidelberg Retina Tomograph III (HRT) and standard automated perimetry (SAP; HFA 640i, Humphrey Field Analyzer) due to the short pre-intervention assessment period, ordinary least squares regression was used to calculate statistically significant linear rates of progression (Pathak *et al.*, 2013; Wang *et al.*, 2013) of mean deviation (MD), visual field index (VFI) and rim area, defined by a negative slope and *P < *0.05. R = right; L = left.

### Study design and randomization

The sequential, ascending, single-dose Storer study design enabled assessment of separate ANX776 dosing cohorts, starting with 0.1 mg, then 0.2 mg, 0.4 mg and finally 0.5 mg. Each cohort comprised two patients with glaucoma and two healthy participants, and a further glaucoma and a healthy subject allocated in reserve in case of adverse events. The design of the study is shown in Supplementary Fig. 3 and Supplementary Table 4.

Following sequential enrolment, subjects were randomly allocated to specific ANX776 dosing positions, as determined by an electronic random allocation system (provided by Sealed Envelopes Ltd, London), and by the time point at which they joined the trial. Initially, six participants were recruited per cohort dose [two patients with glaucoma and two healthy, plus reserve glaucoma (one patient) and healthy (one subject)]. Escalation to the next incremental dose occurred in the absence of adverse events, and on agreement of an Independent Data Monitoring Committee (IDMC). Randomization was performed by the study team, who entered the required data in the system and retrieved an allocation number, which determined the dose to be administered and the patient order in each dosing cohort. In accordance with random allocation, the dose was subsequently administered by the study investigator, according to the Storer design detailed in Supplementary Table 4. One subject was dosed per day with a minimum time period between dosing of 72 h between positions 1 and 2 in a cohort and also at dose escalation, and of 24 h between all other positions in the cohort.

### ANX776 structure and characterization

The chemical structure of ANX776 is shown in Supplementary Fig. 6. ANX776 consists of a variant of human annexin 5 (ANX) named rhAnnexin V128 (Anx V128), which allows a single covalent bond to be made between the maleimide form of the fluorescent dye Dy776-maleimide (Dy-776-mal), and the cysteine residue of Anx V128. The fluorescent properties of ANX776 are due to the conjugated dye, which has an excitation/emission of 771/793 nm, in the near infrared region. This is similar to the absorption and emission spectrum of indocyanine green, a dye that is used frequently in retinal angiography in ophthalmology (Keane and Sadda, 2010). The biological activity of ANX776 has been confirmed using *in vitro* assays (Supplementary Fig. 6), a GLP red blood cell displacement assay (Tait *et al.*, 2004) and several preclinical *in vivo* studies (Supplementary Table 1).

### Intervention

A single injection of one dose of ANX776 was administered intravenously to each subject, following which retinal imaging was performed to visualize ANX776-positive fluorescent cells. ANX776 was formulated at a single strength (0.2 mg/ml) so that the cohort dosage was varied by volume; hence 0.5, 1.0, 2.0 and 2.5 ml were injected from the ANX776 vial to give the 0.1, 0.2, 0.4 and 0.5 mg cohorts, respectively. Assessments of safety and clinical effects/efficacy (secondary outcome) were made at regular intervals (pre-dose, at 5, 15, 30, 60, 120, 240 and 300 min and 30 days after administration).

Images were acquired from all subjects with a confocal scanning laser ophthalmoscope (HRA+OCT Spectralis, Heidelberg Engineering) set to ICGA infrared fluorescence settings (diode laser 786 nm excitation; photodetector with 800 nm barrier filter), after pupillary dilatation (1% tropicamide and 2.5% phenylephrine). Baseline reflective (to ensure focusing on the level of the RNFL was achieved) and infrared autofluorescent images were acquired prior to ANX776 administration, and then during and after ANX776 injection at 15, 30, 60, 120, 240 and 360 min, with fovea-centred images including the whole macula and the optic disc per eye per time point (Supplementary Video 1).

For each time point, sequences of 100 frames were averaged using the manufacturer’s eye tracking system to obtain the highest signal-to-noise ratio, with an image resolution of 1536 × 1536 pixels (30° field of view, at 10 µm/pixel with an OCT axial resolution of 3.9 µm), corresponding to an average width of 8.87 ± 0.28 mm per image, depending on patients’ refraction. The photodetector sensitivity was adjusted to an absolute value of 107 for all images to maintain comparability. Imaging was not possible in one eye of one of the glaucoma patients due to corneal disease; this eye was excluded from the analysis.

### Clinical assessments

All subjects underwent a complete eye examination at each study visit, including best-corrected visual acuity, slit-lamp biomicroscopy, intraocular pressure measurement with Goldmann applanation tonometry, gonioscopy, dilated funduscopic examination with a 78-diopter (D) lens, Heidelberg Retina Tomograph III, OCT and standard automated perimetry. Other assessments included adverse medical event queries, medical and ophthalmic histories and fundus examination with auto-fluorescence (488 and infrared).

All subjects were required to attend three visits when all above tests were repeated: a qualification (after screening), a procedural and follow-up (at 30 days); glaucoma subjects were in addition subsequently reviewed at further follow-up visits, as part of the standard of care, up to 16 months after DARC.

### Safety and tolerability

Only one subject was dosed with ANX776 per day, with a minimum of 72 h between dosing of the first and second participant in any dose cohort. Subjects were required to stay in the hospital and were carefully monitored for adverse events using the Common Terminology Criteria for Adverse Events (CTCAE v4.0), with vital signs being taken at regular intervals, for a period until 6 h after ANX776 administration. Further safety was monitored by a 24-h post-treatment telephone call and finally at 30 days (Visit 3).

### Pharmacokinetic assessments

Serum samples were taken pre-dose, and 5, 15, 30, 60, 120 and 300 min after administration of ANX776 and processed for detailed pharmacokinetic analyses that were performed with the use of a validated sandwich ELISA assay in a GCLP-accredited laboratory. The assay uses a captive antibody specific for human annexin V-128 and a rabbit anti-DY776 secondary antibody. Samples were processed and analysed in a masked fashion.

### DARC analysis

Anonymized retinal images, grouped per subject, were then processed and analysed with blinding to both dosage and subject’s glaucoma status. For each subject, baseline retinal images were aligned to subsequent images per time point using an affine transformation (Modat *et al.*, 2014), followed by a non-rigid transformation (Modat *et al.*, 2010) to compensate for the presence of non-linear optical distortions. Images were then illumination-matched to each other by estimating the differential intensity inhomogeneity to the mean intensity over all time points (Lewis and Fox, 2004). The illumination-corrected baseline image was subtracted from each subsequent image to remove large non-enhancing features such as the retinal vessels and the optic disc (Supplementary Fig. 5).

Fluorescent ANX776-positive spots were automatically computed for each image, at all time points for each subject. To detect these spots in the processed images, a template matching approach was used with a Gaussian kernel with seven pixels standard deviation as a DARC spot template, convolved with each image (Brunelli, 2009). A positive, definite spot was identified by any pixel location with a template matching response above 0.5 in at least two time points. To avoid repeated counting of the same spot at different time points, the DARC count was defined as the first appearance of new, unique individual ANX776-labelled spots. The DARC count was used to assess the efficacy of the technique and in the comparison of healthy controls to glaucoma patients.

### Statistical analysis

All the safety and tolerability and PK analyses included subjects who received ANX776. One eye of one glaucoma patient could not be imaged due to corneal disease, so was excluded from the efficacy analysis. One healthy subject on review of all patients and ahead of the retinal imaging analysis, was found to have bilateral suspicious baseline glaucomatous visual fields and both eyes were excluded from the efficacy analysis. Descriptive statistics were used to summarize the baseline characteristics findings. DARC counts and pharmacokinetics data were determined by means of a one-way ANOVA, with two-way ANOVA being applied to assess the influence of glaucoma status, dose of ANX776 and time. To ascertain whether DARC counts were higher in the eyes of patients with glaucoma, the total counts were compared allowing for the effect of different ANX776 doses, using Stata/IC 11.2 for Windows. The DARC counts were log transformed after adding 1; a log transformation (i.e. a multiplicative model) was used because of the increase in standard deviations of counts with the count size and the recognition that counts cannot be negative. Dose was entered into this analysis as a blocking factor. Spearman’s correlation was used to compare the DARC count to all parameters listed in Table 4. *P*-values < 0.05 were considered statistically significant.

## Results

### Subject demographics

A total of 33 subjects were pre-screened according to the inclusion/exclusion criteria in Supplementary Table 2, from which eight healthy participants and eight patients with progressing glaucoma underwent DARC (Supplementary Figs. 3 and 4). Of note, although a separate group of four patients with non-arteritic ischaemic optic neuropathy had originally been planned as positive controls (one per dosing cohort) none were successfully recruited and since a positive signal was seen with DARC even at low doses, the IDMC, Trial Management Group and sponsor decided to complete the study without them (Supplementary Fig. 3).

Baseline characteristics of these 16 subjects are presented in Table 2 and Supplementary Table 7. Glaucoma patients had significantly increased cupping (*P < *0.0001; mean cup-to-disc ratio 0.53 ± 0.12, range 0.3–0.7 in 15 eyes) and elevated intraocular pressures (*P = *0.033, mean intraocular pressure 15.4 ± 2.1, range 14–21 mmHg) compared to healthy subjects (mean cup-to-disc ratio 0.30 ± 0.06, range 0.2–0.4 in 14 eyes, mean intraocular pressure 13.9 ± 2.1, range 10–16 mmHg), and were diagnosed as either glaucoma suspects or early glaucoma (mean deviation −1.81 ± 1.79 range −5.7–1.05 dB). Over a period of pre-intervention assessment (mean 7.3 ± 1.8 months), structural (OCT and/or Heidelberg Retina Tomograph) and visual field progression [where progression was defined by a significant (*P < *0.05) negative slope] was recorded in at least one eye of each patient, as summarized in Table 1 and detailed in Supplementary Table 8.

**Table 2:** Baseline demographic characteristics of subjects

	Glaucoma	Normal	*P*-value
Sample size	8	7	
Age (years)	53.5 ± 4.9	49.1 ± 8.39	NS
Gender			
Male	5 (62.5%)	5 (75%)	
Female	3 (37.5%)	2 (25%)	
Ethnicity			
Caucasian	5 (62.5%)	3 (40%)	
Black	3 (37.5%)	0	
Asian	0	4 (60%)	
Weight (kg)	76.9 ± 13.0	85.1 ± 16.2	NS
Body mass index	25.7 ± 2.5	28.8 ± 4.0	NS
Systolic blood pressure (mmHg)	127.3 ± 9.2	140.8 ± 13	NS
Diastolic blood pressure (mmHg)	81.0 ± 13.4	85.8 ± 7.8	NS
Heart rate (beat/min)	70.5 ± 10.6	73.1 ± 9.9	NS
Respiration rate (breaths/min)	20.5 ± 1.5	20.8 ± 2.1	NS
Visual acuity			
Right	0.03 ± 0.07	0.01 ± 0.07	NS
Left	0.04 ± 0.07	0.04 ± 0.07	NS
Corneal pachymetry			
Right	542.8 ± 39.5	561 ± 29	NS
Left	548.5 ± 36.5	557 ± 29	NS
Cup/disc ratio			
Right	0.53 ± 0.09	0.30 ± 0.06	**<0.05**
Left	0.54 ± 0.14	0.30 ± 0.06	**<0.05**
Mean intraocular pressure (mmHg)^a^			
Right	15.54 ± 2.02	13.90 ± 2.17	**<0.05**
Left	15.08 ± 1.53	14.05 ± 1.89	**<0.05**
Mean deviation visual fields (MD)			
Right	−1.48 ± 1.49	0.06 ± 2.77	**<0.05**
Left	−2.10 ± 2.09	−0.45 ± 1.12	**<0.05**

Values are mean ± SD.

^a^Five subjects in the glaucoma group were on intraocular pressure lowering medication.

Subjects were consecutively enrolled and randomized to ANX776 dosing. As no serious adverse events were seen at any dose, each ANX776 dose cohort consisted of two patients with glaucoma and two healthy participants.

### Visualization of individual retinal cell apoptosis with DARC

**Figure 1:**
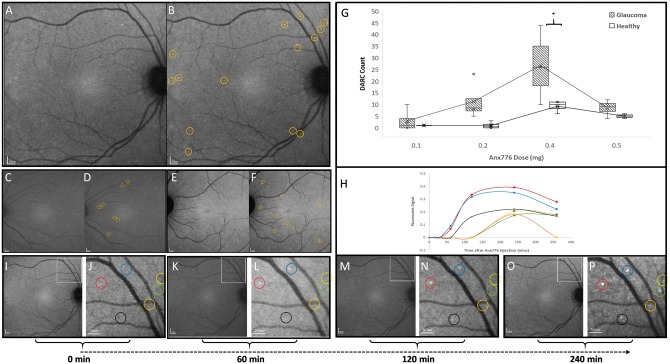
**DARC counts are increased in affected glaucoma patients compared to healthy controls.** ANX776 injections revealed single neuronal cell apoptosis in the retina of study subjects. Representative retinal images are shown from glaucoma patients following intravenous injections of 0.4 (**A** and **B**), 0.2 (**C** and **D**) and 0.5 (**E** and **F**) mg ANX776 at 240 minutes. Panels show unmarked (**A**, **C** and **E**) and marked (**B**, **D** and **F**) ANX776-positive spots with yellow rings highlighting individual spots. DARC counts were defined as new, unique individual ANX776-labelled spots, at their first appearance in the retina. Analysis of DARC counts in glaucoma and healthy controls for each ANX776 dosing cohort showed that at each dose, the number of DARC spot counts was consistently higher in glaucoma patients compared to healthy controls, and this reached significance at the 0.4 mg (*P < *0.005) dose (**G**). The spread of the individual data points is shown in Tukey’s box plots (**G**). Horizontal lines indicate medians and interquartile ranges with the continuous line across doses showing the means. Asterisks indicate the level of significance by Bonferroni multiple comparison test between groups (*P < *0.01) with two-way ANOVA across the doses showing a significant effect of glaucoma status (*P = *0.0033) and time point (*P = *0.0011). Multivariable analysis indicated that the total DARC count across 6 h was 2.37-fold higher in patients with glaucoma (95% CI: 1.4–4.03, *P = *0.003) at any dose. Different fluorescent intensity profiles were seen for individual labelled spots (**H–P**). Low (**I**, **K**, **M** and **O**) and high (**J**, **L**, **N** and **P**) magnification (scale bars indicated) retinal images at different time points are shown from the same patient as in **A** at baseline (**I** and **J**, 0 min), 60 (**K** and **L**), 120 (**M** and **N**) and 240 (**O** and **P**) min. Marked, colour-coded spots are shown in adjacent panels (**J**, **L**, **N** and **P**) with fluorescent intensity profiles illustrated in **H**, identified by corresponding coloured lines.

DARC spots, identified as ANX776 positive-labelled cells, were visualized with fluorescent imaging as hyperfluorescent spots on the retina measuring between 12 and 16 µm diameter (Supplementary Video 1 and Fig. 1). The retinal area visualized in each image was 78.73 ± 5.04 mm^2^. Figure 1A–F shows the typical appearance of DARC spots at different concentrations of ANX776, with a significant increase in DARC activity at the 0.4 mg dose (*P < *0.01, Fig. 1G). Individual DARC spots had different fluorescent signal profiles over time, as illustrated in Fig. 1H–P. DARC spots were at the level of the retinal ganglion cell layer, as judged by focusing in the reflective mode of the confocal scanning laser ophthalmoscope, with visualization of hyper-reflective nerve fibre bundles (Supplementary Figs 9 and 10).

### Comparison of DARC in patients with glaucoma and healthy subjects

All doses showed a higher DARC count in glaucoma patients compared to healthy controls with two-way ANOVA across the doses showing a significant effect of glaucoma status (*P = *0.0033) and time point (*P = *0.0011). Multivariable analysis indicated that the total DARC count across 6 h was 2.37-fold higher in patients with glaucoma [95% confidence interval (CI): 1.4–4.03, *P = *0.003], at any dose. The ‘-fold’ changes were 2.36, 1.68, 1.50, 1.37, 1.84 and 1.87, respectively, at 15, 30, 60, 120, 240 and 360 min, with upper 95% CIs of ≥ 2.34 and *P*-values of 0.009, 0.051, 0.09, 0.23, 0.01 and 0.02, respectively for glaucoma versus healthy participants.

The DARC count was found to be significantly correlated with decreased central corneal thickness (Spearman’s R = −0.68, *P = *0.006) and increased cup–disc ratios (Spearman’s R = 0.47, *P = *0.038) in glaucoma patients. It was also positively correlated with age (Spearman’s R = 0.77, *P = *0.001) in healthy control subjects.

### Correlation of DARC with disease activity

**Figure 2:**
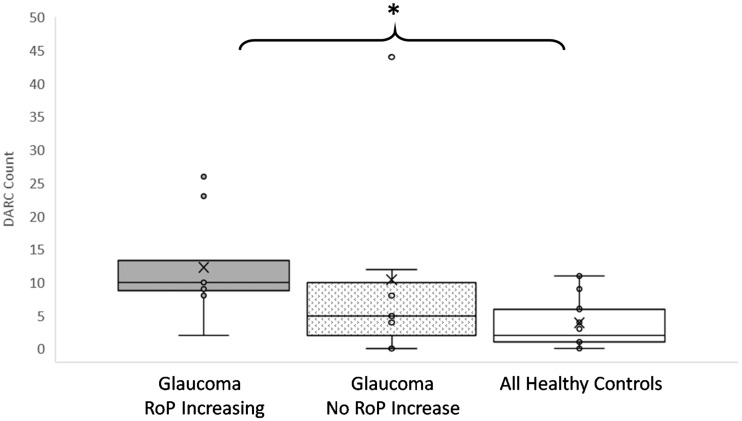
**DARC counts are significantly increased in glaucoma patients with increasing rates of progression compared to healthy controls.** The rate of progression (RoP) was calculated for all parameters at baseline and follow-up for all glaucoma patients, where an increasing rate of progression was computed from the difference between follow-up and baseline significant (*P < *0.05) negative slopes. The DARC count was significantly increased in glaucoma patients with increasing rate of progression in any one parameter, compared to healthy controls, as shown in Tukey’s box plots illustrating individual data points in glaucoma patients with and without increasing rate of progression compared to healthy controls. Asterisks indicate level of significance by Dunn’s multiple comparison test between groups (*P < *0.05) with Kruskal Wallis one-way ANOVA showing statistical significance across three groups (*P = *0.0448). Horizontal lines indicate medians and interquartile ranges with ×’ symbol showing the means, and all individual data points indicated. See also Table 3.

To be eligible for the study, glaucoma patients had to show evidence of progression of disease in at least one of the parameters summarized in Table 1, defined by a significant negative slope in the rate of progression. In addition to calculating rate of progressions at baseline, a *post hoc* assessment was performed at follow-up to obtain final rate of progressions, as shown in Supplementary Table 8. Patients were identified who showed a higher rate of progression at follow-up than at baseline in at least one parameter, as highlighted in red text in Supplementary Table 8 and summarized in Table 3 (increasing rate of progression). Analysis of the DARC count in these patients showed it to be significantly (*P = *0.028) increased compared to healthy controls (Fig. 2 and Table 3).

**Table 3:** DARC counts are significantly increased in glaucoma patients with increasing rates of progression compared to healthy controls

		Number of glaucoma eyes with significant RoP (*P* < 0.05; total *n* = 15)		
Instrument	Parameters	Baseline	Follow-up	RoP increasing
HRT	Rim area	2	3	2
OCT	RNFL 3.5	8	10	3
	RNFL 4.1	4	3	2
	RNFL 4.7	2	3	1
	MRW	3	4	1
SAP	MD	3	5	3
	VFI	0	1	1

MD = mean deviation; MRW = Bruch’s membrane opening minimum rim width; RoP = rate of progression; VFI = visual field index. See also Fig. 2.

### Safety, tolerability and pharmacokinetics

No patients withdrew from the study, and no serious adverse events were reported. Six separate adverse events were described as detailed in Table 4, all of which were mild, self-limiting and probably unrelated to ANX776. There were single cases of: discomfort during phlebotomy, haematoma at cannulation site, influenza, metatarsal inflammation, dizziness and headaches.

**Table 4:** Adverse events

Adverse event	Patients, *n* (%)	Severity^a^	Relationship to IMP^b^	Duration	Glaucoma	Previous history
Discomfort during phlebotomy	1 (6.25)	1	4	<1 min	No	Yes
Haematoma following cannulation	1 (6.25)	1	4	1 day	Yes	No
Influenza	1 (6.25)	1	4	3 days	No	Yes
Metatarsal inflammation	1 (6.25)	1	4	3 weeks	Yes	No
Dizziness	1 (6.25)	1	4	<1 min	No	Yes
Headache	1 (6.25)	1	4	2 h	No	Yes

^a^Severity: 1, mild; 2, moderate; 3, severe; 4, life threatening; 5, death.

^b^Relation to study drug: 0, definitely; 1, probably; 2, possibly; 3, unlikely; 4, not related; 5, not assessable.

IMP = investigational medicinal product.

**Figure 3:**
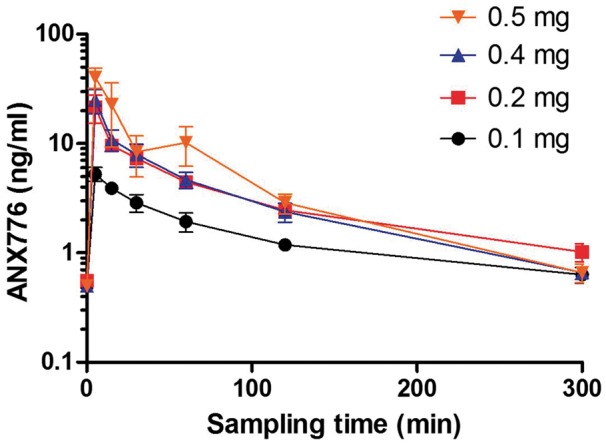
**ANX776 pharmacokinetics and pharmacodynamics.** The mean serum concentration of ANX776 over time after a single intravenous administration of five different dose levels is shown with standard error bars in healthy and glaucoma cohorts. See also Table 5.

ANX776 was rapidly absorbed and eliminated after intravenous administration (Fig. 3 and Table 5). Pharmacokinetic results showed that exposure to ANX776 was dose-dependent with no accumulation. C_max_ (maximum serum concentration) increased proportionally with increasing doses as did the AUC (area under the serum concentration time curve to the 5-h collection time). Mean (and median) C_max_ were 5.5 (5.6), 21.6 (19.2), 25.8 (25.6) and 40.9 (39.4) ng/ml, for the 0.1, 0.2, 0.4 and 0.5 ANX776 mg cohorts, respectively. The mean T_max_ (time to C_max_) was 6.875 min, and median 5 min, consistent with the short half-life (T_½_) which was 36.4, 18.8, 20.7 and 20 min for the 0.1, 0.2, 0.4 and 0.5 mg cohorts, respectively. ANX776 clearance was correspondingly high (mean 354.75, median 372.5 ml/min). Analysis of any differences between subject groups showed that except at the lowest 0.1 mg dose, there was no significant difference between glaucoma and healthy subjects in any of the pharmacokinetic parameters measured.

**Table 5:** Pharmacokinetic parameters

	Cohort 1 *n = *4	Cohort 2 *n = *4	Cohort 3 *n = *4	Cohort 4 *n = *4
Dose, mg	0.1	0.2	0.4	0.5
C_max_, ng/ml	5.5 (0.5)	21.6 (6.2)	25.8 (5.7)	40.9 (8.7)
Tmax, min	7.5 (2.5)	5.0 (0.0)	7.5 (2.5)	7.5 (2.5)
AUC_0–300 min_, ng/ml	290 (24)	886 (109)	909 (60)	1490 (520)
C_min_, ng/ml	0.6 (0.1)	1.0 (0.2)	0.7 (0.1)	0.7 (0.1)
T_1/2_, min	36.4 (14.8)	18.8 (7.2)	20.7 (9.7)	10.2 (4.9)
K_e_, min^−1^	0.036 (0.015)	0.070 (0.031)	0.098 (0.064)	0.133 (0.050)
Clearance, ml/min	352 (30)	193 (66)	446 (29)	428 (88)

Data are shown as mean (standard error). C_max_ = maximum serum ANX776 concentration after iv administration; T_max_ = time to maximum serum concentration; AUC = area under the serum concentration time curve to the last collection time (300 min); C_min_ = minimum serum ANX776 concentration; T_1/2_ = terminal elimination phase half-life; K_e_ = elimination rate constant. See also Fig. 3.

## Discussion

This proof-of-concept study demonstrates that visualization of individual retinal cell apoptosis is possible in the human retina. Using DARC technology with a fluorescent apoptosis marker, ANX776, we have identified a significantly increased level of retinal cell apoptosis in glaucoma patients compared to healthy controls. The DARC count was significantly greater in patients with progressing glaucomatous disease. Finally, we found ANX776 to be safe and well-tolerated, with a short half-life.

As far as we are aware, this is the first time individual apoptosing neuronal cells have been visualized *in vivo* in humans. Previous use of annexin 5 in patients has been when it was labelled with Technetium-99 m; however, nuclear medicine techniques are unable to resolve microscopic processes due to insufficient spatial resolution, and show instead regional areas of apoptotic activity (Supplementary Table 11) (Vangestel *et al.*, 2011). The visualization of distinct fluorescently-labelled neuronal cells is currently possible only in the eye, due to its unique optical properties, providing a promising opportunity to identify and assess neurodegenerative disease.

RGC apoptosis occurs early in glaucomatous neurodegeneration (Garcia-Valenzuela *et al.*, 1995; Quigley *et al.*, 1995; Kerrigan *et al.*, 1997; Kerrigan-Baumrind *et al.*, 2000; Cordeiro *et al.*, 2004; Guo *et al.*, 2007; Quigley, 2011). Its presence has been clearly documented by different methods and investigators (Garcia-Valenzuela *et al.*, 1995; Quigley *et al.*, 1995; Kerrigan *et al.*, 1997; Okisaka *et al.*, 1997; Nickells, 1999; Kerrigan-Baumrind *et al.*, 2000; Tatton *et al.*, 2001; Reichstein *et al.*, 2007), with some even suggesting that ‘Annexin-V can be used to specifically detect apoptotic RGCs … . in glaucomatous … retina’ (Reichstein *et al.*, 2007). Using DARC and retrograde labelling *in vivo,* we have confirmed by histological analysis (Cordeiro *et al.*, 2004, 2010), that fluorescent ANX labels apoptosing RGCs in different glaucoma experimental models (Supplementary Table 1) (Cordeiro *et al.*, 2004; Guo *et al.*, 2005, 2006). Building on our preclinical data, we decided to investigate glaucoma clinically as the neurodegenerative condition to be first assessed using DARC, in a proof-of-concept study.

This study revealed significantly elevated DARC counts in glaucoma patients compared to healthy subjects, suggesting that DARC could be used to identify abnormal retinal neurodegenerative activity. Glaucoma patients in this study had early glaucoma (visual field mean deviation: −1.81 dB) but with significant progression in any parameter in at least one eye. These patients progressed in a short time, with mean baseline and final follow-up of 7.3 and 13.0 months, respectively. Few studies have looked at short follow-up times, although reproducibility studies assessing test–retest variability and coefficient of variations have been performed in similar time frames (Wolf-Schnurrbusch *et al.*, 2009; Wang *et al.*, 2013). The rate of visual field progression is used in glaucoma as a marker of neurodegeneration, especially with respect to neuroprotective treatments (Krupin *et al.*, 2011), as it reflects the rate of RGC loss (Harwerth and Quigley, 2006). The visual field index trend analysis is normally based on five tests over 2 years or more (Bengtsson *et al.*, 2009), with early glaucoma corresponding to a mean visual field index trend of −0.89 to −0.83% per year (Cho *et al.*, 2012; Aptel *et al.*, 2015). Recently, however, the rate of RNFL loss in OCT has been shown to be predictive of visual field loss (Yu *et al.*, 2016). An interesting *post hoc* finding in the present study has been the significant relationship of a high DARC count being predictive of increased rates of progression, suggesting that DARC could potentially be prognostic of neurodegenerative activity.

Based on glaucomatous RGC loss being on average 4% (Zeyen, 1999; Hirooka *et al.*, 2016) compared to that attributed to normal ageing (0.4%), (Jonas *et al.*, 1992; Harman *et al.*, 2000; Neufeld and Gachie, 2003; Harwerth *et al.*, 2008), the number of RGCs lost per year in glaucomatous disease has been estimated to be between 28 000 (Hirooka *et al.*, 2016) and 33 000 (Medeiros *et al.*, 2012), or between 77 and 90 per day in the whole retina. Assuming the 30° lens of the OCT Spectralis used in this study visualizes 30–52 % of the total RGC, and the DARC count represents the daily RGC death within that field of view (assuming all cell death occurs through apoptosis or necrosis, and therefore labelled as ANX776-positive), then our findings of the maximal DARC counts in the glaucoma 0.4 mg ANX776 cohort would be in the predicted range. This will clearly need further study, as will the correlation of DARC with reduced central corneal thickness, controversially cited as a risk factor for developing glaucoma (Gordon *et al.*, 2002; Brandt *et al.*, 2012).

Another interesting finding in this study is the positive correlation of age with the DARC count in healthy subjects. Apoptosis has been associated with ageing (Kujoth *et al.*, 2005), but is also an established risk factor for incidence and progression in glaucoma (Leske *et al.*, 2007; Chauhan *et al.*, 2008). Again, larger trials are needed to further investigate this, and to study its application to other neurodegenerative diseases (Supplementary Table 1) (Cordeiro *et al.*, 2004, 2010; Normando *et al.*, 2013).

The method of template matching is routinely used for tracking cells in microscopy (Brunelli, 2009; Adanja *et al.*, 2010; Dewan *et al.*, 2011), with the same principles applied to allow us to analyse single cells *in vivo* longitudinally in this study. This enabled the analysis of the DARC count to be possible as it consisted purely of the appearance of new, ANX776 positively labelled cells.

Our pharmacokinetic studies indicate that ANX776 is rapidly absorbed and distributed, which limits the peak plasma levels and the potential for acute adverse reactions. The terminal elimination half-life (ranging from 10 to 36 min) is similar to that reported of radiolabelled Anx V128 experimentally (Benali *et al.*, 2014), which is currently being assessed in patients (NCT02182609). The optimal dosing concentration appears to be 0.4 mg, as this was when the peak level of DARC counts was seen. Interestingly, this is within the range of the radiolabelled Anx A5 clinical trials, as summarized in Supplementary Table 11. At 0.4 mg, the half-life of ANX776 is 20.7 min, with a clearance of 29 ml/min. This again fits with published literature, which suggests that the Anx V128 mutation provides an 88% lower renal uptake than wild-type Anx A5, with a faster clearance (Benali *et al.*, 2014). In addition, there is good evidence that the single-site labelling of Anx V128 is associated with an increased sensitivity of detection of apoptotic cells (Tait *et al.*, 2006). The half-life range in our study was dependent on dosage, with the longest half-life at the lowest dose of 0.1 mg. Commonly, half-life increases with concentration due to zero order kinetics; however, as with vitamin C, reductions in half-life with increasing concentrations can be seen with second order kinetics. This may also be explained by the hypothesis that at the lowest doses, a greater proportion of ANX776 may be binding to phosphatidylserine with little left to be eliminated. At higher doses the effect would be less, and elimination would reflect the unbound fraction of ANX776.

Despite these promising results, it is important to recognize that these are only preliminary. Like any new technology, DARC will need robust testing if it is to be successfully validated (Kenis *et al.*, 2010; Henry and Hayes, 2012). However, these results demonstrate translation from experimental studies where DARC has been used to assess treatment efficacy in addition to disease activity, opening the door for it to be considered as a companion diagnostic endpoint in the indications already investigated experimentally. Further studies will be needed to validate these initial findings, but these encouraging data are useful in the upcoming clinical studies, where DARC will be assessed not only in glaucoma, but also in age-related macular degeneration, optic neuritis, and Alzheimer-related disease.

In conclusion, this study demonstrates that using ANX776 and DARC, retinal cell apoptosis can be identified in the human retina with increased levels of activity in glaucomatous neurodegenerative disease. As far as we are aware, this is the first time in humans that individual neuronal cell apoptosis has been visualized *in vivo*, in real time. The DARC count appears predictive of progressive disease, indicating its possible use as a surrogate marker in glaucoma. Further studies will be needed to validate these initial findings, but this encouraging data is useful in the upcoming phase 2 studies where DARC will be assessed not only in glaucoma, but also in age-related macular degeneration, optic neuritis, and Alzheimer-related disease; this may provide additional information regarding the potential of DARC in evaluating disease activity and treatment efficacy in other neurodegenerative conditions.

## Supplementary Material

Supplementary Data

## Abbreviations

DARC: detection of apoptosing retinal cells
OCT: optical coherence tomography
RGC: retinal ganglion cell
RNFL: retinal nerve fibre layer
