# Supplementary material for: Real-time imaging of single neuronal cell apoptosis in patients with glaucoma
Source: Brain. 2017 Apr 26;140(6):1757–67. doi: 10.1093/brain/awx088 (PMC5445254; doi:10.1093/brain/awx088)
Supplement: Supplementary Data [file awx088_Supp.zip › awx088-suppl_data/brain-2016-02139-File006.pdf]

Supplementary Appendix

This appendix has been provided by the authors to give readers additional information about their work.

Supplement to: Cordeiro MF, Normando EM, Cardosa JG et al.

Real-time imaging of single neuronal cell apoptosis in patients with glaucoma

Table of Contents

Real-time imaging of single neuronal cell apoptosis *in vivo* for patients with glaucoma............................ 1
Table S1: Experimental Studies Measuring Disease Activity and Treatment Efficacy with DARC........... 3
Table S2: - Inclusion/Exclusion Criteria 8
Figure S3 Consort Diagram.. 10
Table S4 Storer Design 11
Figure S5: Retinal imaging Flowchart of Analysis Stages 12
Figure S6: Chemical Structure of ANX776 and In Vitro Assays 13
Table S7: Medical Characteristics 16
Table S8: Rates of Progression of Glaucoma Patients at Baseline and Final Follow-up 17
Figure S9: DARC Fluorescent Images at in Healthy and Glaucoma Eyes... 19
Figure S10: DARC Fluorescent Images at different ANX776 Doses in Glaucoma Eyes 20
Figure S11: Development of Anx776 Positive Spots over Time 21
Table S12: Summary of Clinical Trials with Annexin 5 22
Table S13: Supplementary Statistical details 32
Video S14: Supplementary Procedural Video 33
References 34

Table S1: Experimental Studies Measuring Disease Activity and Treatment Efficacy with DARC

Type of Study	Report	Short Title of Study	Species (model)	Label	Findings	Disease Activity or Treatment Efficacy
Proof of Concept	(Cordeiro *et al.*, 2004)	Real time imaging of single nerve cell apoptosis in retinal neurodegeneration. Proceedings of the National Academy of Science	*Rat* *Glaucoma* *Optic neuropathy*	Anx with Alexa-Fluor 488 tag	Study confirmed that neuronal apoptosis in the eye of a living animal could be observed in real time and at a cellular level using fluorescent labelled annexin, and results were validated histologically.	Disease activity
Proof of Concept	(Guo *et al.*, 2005)	Retinal ganglion cell apoptosis in glaucoma is related to intraocular pressure (IOP) and IOP-induced effects on extracellular matrix.	*Rat* *Glaucoma*	Anx with Alexa-Fluor 488 tag	The results demonstrated that RGC apoptosis in glaucoma correlates strongly with elevated IOP and is significantly associated with IOP-induced changes in specific ECM components in the RGC layer.	Disease activity
Proof of Concept	(Guo *et al.*, 2006)	Assessment of neuroprotective effects of glutamate modulation on glaucoma-related retinal ganglion cell apoptosis in vivo.	*Rat* *Glaucoma*	Anx with Alexa-Fluor 488 tag	RGC apoptosis was induced in rats by staurosporine (SSP) treatment. Single agents MK801, ifenprodil, or LY354740, or MK801 and LY354740 combined, were administrated intravitreally at different doses. Eyes were imaged in vivo using fluorescent labelled annexin and the results confirmed histologically. This novel SSP model was validated as a useful tool for screening neuroprotective strategies in vivo showing treatment efficacy with DARC	Treatment efficacy

Type of Study	Report	Short Title of Study	Species (model)	Label	Findings	Disease Activity or Treatment Efficacy
Proof of Concept	(Maass *et al.*, 2007)	Assessment of rat and mouse RGC apoptosis imaging in vivo with different scanning laser ophthalmoscopes	*Rat and Mouse* *Glaucoma*	Anx with Alexa-Fluor 488 tag	Fluorescent points (FPs) used as a measure of RGC apoptosis in vivo were detected in the mouse eye but only with the HRAII and not the Zeiss cSLO. The HRAII was able to detect 62% more FPs in rat than the Zeiss cSLO. Both cSLOs showed peak FP counts at the 5- to 10-microm range in rat and mouse. Maximal FP counts were detected in the superior and superior temporal regions in the rat, with no obvious pattern of distribution in the mouse. The HRAII was found to have more FP correspondence with histologically identified apoptosing RGCs.	Disease activity
Proof of Concept	(Guo *et al.*, 2007)	Targeting Amyloid-ß in glaucoma treatment.	*Rat* *Alzheimer's* *Glaucoma*	Anx with Alexa-Fluor 488 tag	The study showed that Amyloid-ß colocalizes with apoptotic retinal ganglion cells (RGC) in experimental glaucoma and induces significant RGC apoptosis in vivo in a dose- and time-dependent manner. It was demonstrated that targeting different components of the Amyloid-ß formation and aggregation pathway can effectively reduce glaucomatous RGC apoptosis in vivo, with DARC spot counts being the end point assessing response to therapy. Finally, combining treatments (triple therapy) is more effective than monotherapy. The monitoring technique used involved labelled annexin.	Disease activity & Treatment efficacy
Proof of Concept	(Borrie *et al.*, 2008)	Diabetic Retinal Neurodegeneration: In vivo Imaging of Retinal Ganglion Cell Apoptosis	*Mouse* *Diabetes/transgenic*	Anx with Alexa-Fluor 488 tag	Increased retinal cell apoptosis with DARC was seen in transgenic compared to age-matched controls	Disease activity

Type of Study	Report	Short Title of Study	Species<br><br>(model)	Label	Findings	Disease Activity<br><br>or<br><br>Treatment Efficacy
Proof of Concept	(Schmitz-Valckenberg *et al.*, 2008)	Real-time in-vivo imaging of retinal cell apoptosis after laser exposure.	*Rat*<br><br>*Laser/retinal degeneration*	Anx with IR dye 800	DARC revealed different profiles of retinal cell apoptosis at low exposures (<300 ms and <300 mW) compared to higher exposures (>300 ms and >300 mW) mainly at inner retinal layers. Dose-dependent effects on spot density and positive correlation of spot density between lesion size ($P < 0.0001$) and retinal elevation ($P < 0.0001$) were demonstrated. Histology confirmed the presence of apoptosing retinal cells in the inner nuclear and the ganglion cell layers.	Disease activity
Proof of Concept	(Schmitz-Valckenberg *et al.*, 2009)	In-vivo imaging of retinal cell apoptosis following acute light exposure.	*Rat*<br><br>*Light toxicity/retinal degeneration*	Anx with Alexa-Fluor 488 tag	Directly after light exposure, no pathological retinal changes were observed by DARC imaging (fluorescent labelled annexin). However, retinal flattening and the development of apoptosis within the irradiated retina occurred 1 day later and following dark adaptation. This was confirmed by histological analysis, which clearly showed photoreceptor apoptosis.	Disease activity
Proof of Concept	(Cordeiro *et al.*, 2010)	Imaging multiple phases of neurodegeneration: a novel approach to assessing cell death in vivo.	*Rat*<br><br>*Glaucoma*<br><br>*Mouse*<br><br>*Transgenic Alzheimer*	Anx with Alexa-Fluor 488 tag or ANX776	Neuroprotective effects of NMDA antagonsit MK801 shown at 3 weeks using DARC in glaucoma model<br><br>DARC detected retinal cell apoptosis in early Alzheimer's disease in transgenic model but importantly an increase in retinal apoptosis induced by oxidative stress (PMA) could be detected as a measure of disease activity	Disease activity<br><br>&<br><br>Treatment efficacy

Type of Study	Report	Short Title of Study	Species (model)	Label	Findings	Disease Activity or Treatment Efficacy
Proof of Concept	(Normando *et al.*, 2013)	Imaging Dry AMD	*Mouse* *Retinal degeneration/Hypoxia*	Anx with Alexa-Fluor 488 tag	Photoreceptor apoptosis identified histologically	Disease activity
Proof of Concept	(Galvao *et al.*, 2014)	Unexpected Low Dose Toxicity of the Universal Solvent, DMSO	*Rat* *Glaucoma*	ANX776	The study was to demonstrate that DMSO induced retinal apoptosis at low concentrations, with toxicity detected at levels >1%v/v using Anx A5.	Disease activity
Proof of Concept	(Salt *et al.*, 2014)	Effect of the Aβ aggregation modulator MRZ-99030 on retinal damage in an animal model of glaucoma.	*Rat* *Beta-Amyloid/Alzheimer's* *Glaucoma*	Anx with Alexa-Fluor 488 tag	An anti-abeta agent was shown to be effective using DARC to monitor the degree of RGC apoptosis occurring, indicating that this method of RGC detection was suitable for testing treatment efficacy	Treatment efficacy
Proof of Concept	(Guo *et al.*, 2014)	Direct optic nerve sheath (DONS) application of Schwann cells prolongs retinal ganglion cell survival in vivo.	*Rat* *Optic neuropathy*	Anx with Alexa-Fluor 488 tag or ANX776	The study was an investigation of a new method of delivery of Schwann cells for reversing optic neuropathy and used DARC to visualise RGC apoptosis as an outcome of successful rescue	Treatment efficacy
Proof of Concept	*(Galvao et al., 2015)*	Adenosine A3 receptor activation is neuroprotective against retinal neurodegeneration	*Rat* *Optic neuropathy*	Anx with Alexa-Fluor 488 tag	DARC revealed a reduction of RGC apoptosis in the eyes treated with A3 agonist, compared to untreated control in partial optic nerve transection model – assessing its use as neuroprotective endpoint	Treatment efficacy

Type of Study	Report	Short Title of Study	Species<br><br>(model)	Label	Findings	Disease Activity<br><br>or<br><br>Treatment Efficacy
Proof of Concept	(Normando *et al.*, 2016)	The retina as an early biomarker of neurodegeneration in a rotenone-induced model of Parkinson's disease: evidence for a neuroprotective effect of rosiglitazone in the eye and brain.	*Rat*<br><br>*Rotenone model of Parkinson's Disease*	Anx with Alexa-Fluor 488 tag	DARC revealed retinal apoptosis and OCT showed retinal changes before those seen in brain in PD.<br><br>Also showed that DARC could be used to assess response to PD treatment – a new indicator of neurodegenerative activity	Disease activity<br><br>&<br><br>Treatment efficacy
Proof of Concept	Data on file	Assessment of ANX776	*Rat/Mouse*<br><br>*Glaucoma*<br><br>*Optic neuropathy*<br><br>*Alzheimer's (transgenic)*<br><br>*Parkinson's (rotenone and transgenic)*<br><br>*Optic neuritis (LPS)*<br><br>*Rabbit*<br><br>*Retinal Angiogenesis*	ANX776	DARC using ANX776 was able to reveal retinal apoptosis in the rat, mouse and rabbit models of neurodegenerative<br><br>All models also tested with:<br><br>Mitochondrial, oxidative stress, neuroprotective and antiangiogenic agents	Disease activity<br><br>&<br><br>Treatment efficacy

Table S2: - Inclusion/Exclusion Criteria

ALL SUBJECTS	Healthy	Glaucoma
Inclusion criteria<br>• Above 18<br>• Clear optical media in both eyes, as assessed by study investigator<br>• Refractive error with spherical equivalent ≤6D of 6D and BCVA ≥6/24<br>• Have had good fundoscopy with assessment of their optic disc<br>• Participant proven to be able to perform reliable visual fields testing (learning curve for all starters)<br>• Informed consent signed<br>• Women Not of Childbearing Potential.<br>• Male participants: double barrier method	• No ocular or systemic disease confirmed by GP<br>• No evidence of any glaucomatous process either with optic disc, RNFL or visual field abnormalities and with normal IOP's	• No ocular or systemic disease other than glaucoma<br>• Have performed at least 3 HVF, 3 HRT, and 3 OCT<br>• Progression in any of the parameters measured in at least one eye (HVF, OCT, HRT)<br>• Progression was said to be present when a significant rate of progression was established, defined by a negative slope where $p<0.05$ in any of the following specific parameters:<br>- OCT retinal nerve fibre layer (RNFL) measurements at three different diameters from the optic disc (3.5, 4.1, and 4.7 mm), and Bruch's membrane opening minimum rim width<br>- HRT Rim Area<br>- HVF MD and VFI<br>• Diagnosis of glaucoma, glaucoma suspect or ocular hypertensive
Exclusion criteria<br>• Terminal, mental illness, dementia<br>• Presence or history of ocular or systemic uncontrolled/ustable disease<br>• CCT<450 µm or >650µm<br>• Body weight <40kg or >120kg<br>• Chronic neurodegenerative condition<br>• Active antiphospholipid syndrome<br>• Known history of clotting	• History or evidence of glaucoma or clinical suspicion of glaucoma on presentation or IOP ≥ 24 mmHg.<br>• History of systemic vasculitis, collagenosis or ongoing treatment of cancer.<br>• Active uveitis<br>• Evidence of previous	• Uncontrolled IOP >24mmHg.<br>• Angle closure/narrow glaucoma.<br>• Mean deviation with HVF <-12dB.<br>• Unilateral glaucoma<br>• Secondary glaucoma

diseases<br>• Diagnosis of thrombocytopenia, heart valve disease, and livedo reticularis.<br>• Pregnancy and/or lactation<br>• Known allergy to any of the study medication ingredients<br>• Inclusion in a clinical trial within 12 weeks prior to entry into the study<br>• Ocular surgery within the past 3 months<br>• History of retinal laser photocoagulation<br>• Media opacities or retinal pathology or amblyopia limiting visual acuity, HVF or retinal imaging<br>• Subjects expected to need ocular surgery during the study<br>• Any other severe or acute or chronic medical or psychiatric condition	retinal vascular disease	

Figure S3 Consort Diagram

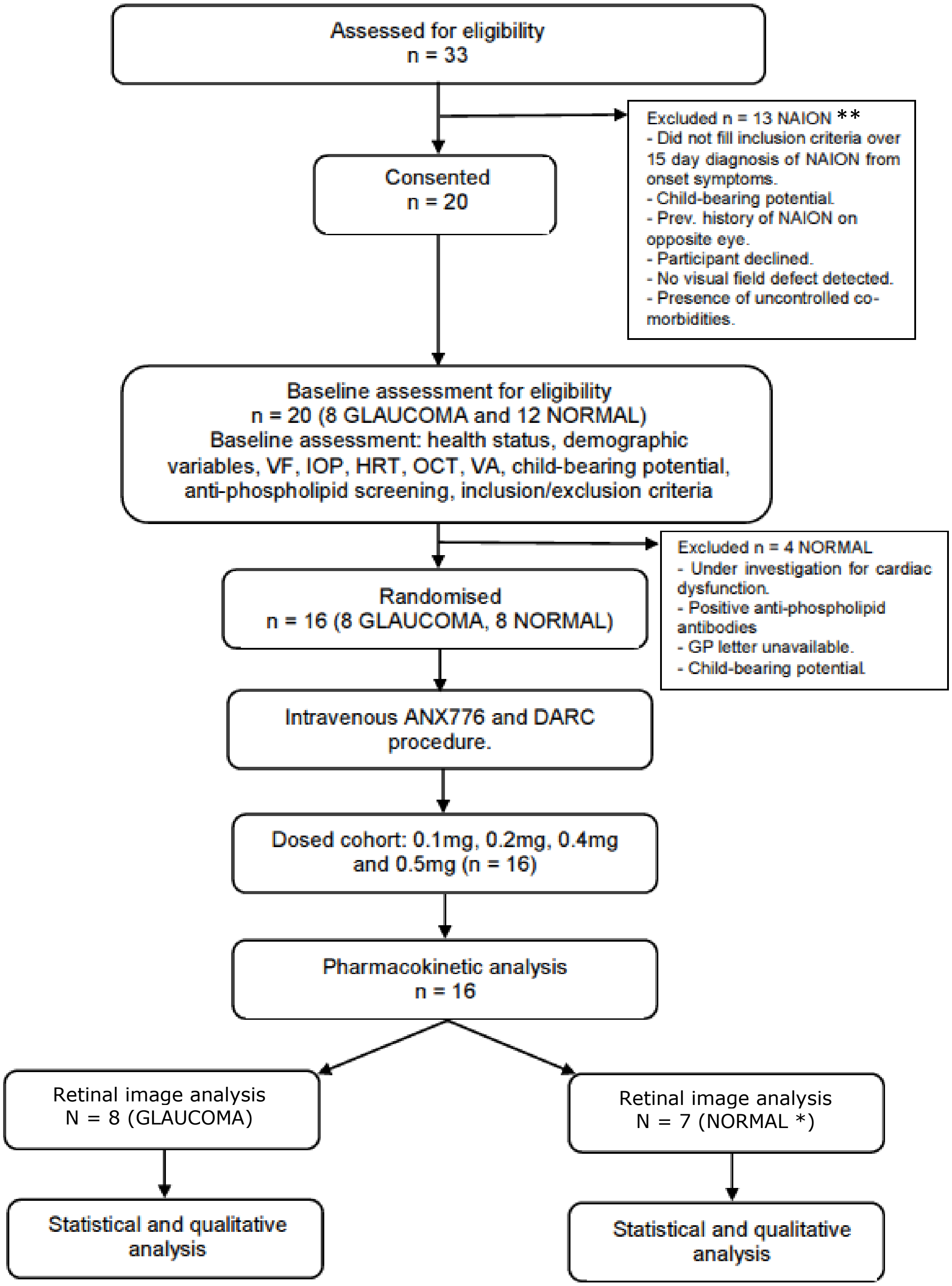

NB: *One NORMAL subject, ahead of the retinal imaging analysis, on review was found to exhibit abnormal baseline visual fields consistent with glaucomatous damage, and was excluded from further analysis.

** As no NAION patients were successfully recruited and since a positive signal was seen with DARC even at low doses, the IDMC, Trial Management Group (TMG) and sponsor decided to complete the study without them.

Table S4 Storer Design

Cohort	No. of subjects	Composition of Cohort	Dose mg	Dose mg/kg	Increment from previous dose
1	4	2 NORMAL<br>2 GLAUCOMA*	0.1	0.0014	-
2	4	2 NORMAL<br>2 GLAUCOMA*	0.2	0.0029	2x
3	4	2 NORMAL<br>2 GLAUCOMA*	0.4	0.0057	2x
4	4	2 NORMAL<br>2 GLAUCOMA*	0.5	0.0071	1.25x

*Randomisation for each dosing cohort included one further “NORMAL” and “GLAUCOMA” held in reserve, in case of adverse events .

Figure S5: Retinal imaging Flowchart of Analysis Stages

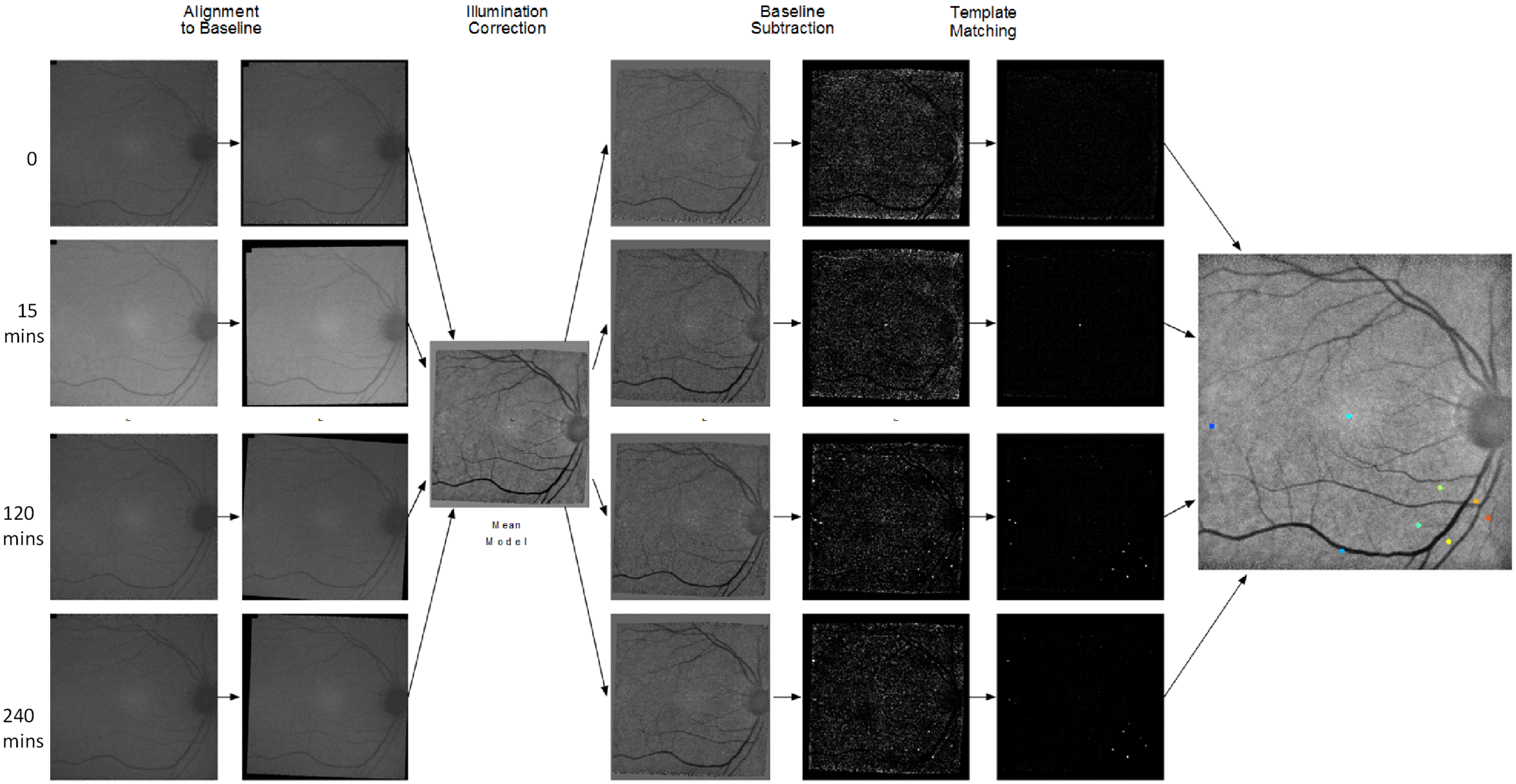

Figure S6: Chemical Structure of ANX776 and In Vitro Assays

Figure 1: Structural formula of ANX776 at the N-terminus, Dy-776-maleimide and the complete amino acid sequence of Anx V128.

A

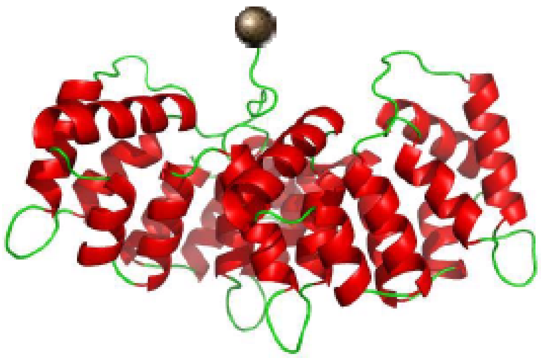

B

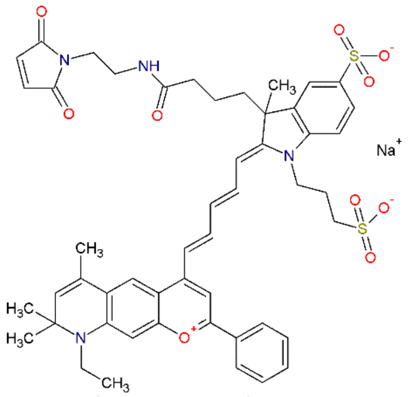

C

AGGCGHAQVLRGTVTDFPGFDERADAETLRKAMKGLG
TDEESILTLLTSRSNAQRQEISAAFKTLFGRDLLDDLKSEL
TGKFEKLIVALMKPSRLYDAYELKHALKGAGTNEKVLTEII
ASRTPEELRAIKQVYEEEYGSSLEDDVVGDTSGYYQRML
VVLLQANRDPDAGIDEAQVEQDAQALFQAGELKWGT
DEEKFITIFGTRSVSHLRKVFDKYMTISGFQIEETIDRETS
GNLEQLLLAVVKSIRSIPAYLAETLYYAMKGA
GTDDHTLIRVMVSRSEIDLFNIRKEFRKNFATSLYSMIKG
DTSGDYKKALLLLSGEDD

D

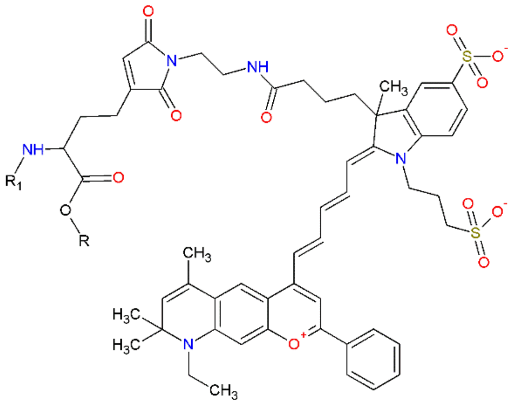

(A) Diagrammatic representation of a Dy-776-mal labelled Anx V128 molecule, with the Dy-776-mal represented by the brown circle and the annexin molecule in green and red. **(B)** The molecular structure of Dy-776-mal. **(C)** The codon optimised sequence of Anx V128, highlighting the added amino acids at the N-terminus containing the cysteine, and the mutated serine. **(D)** The molecular structure of the conjugated fluorescent moiety of ANX776.

Figure 2: Interactions of ANX776 with phosphatidylserine containing membranes

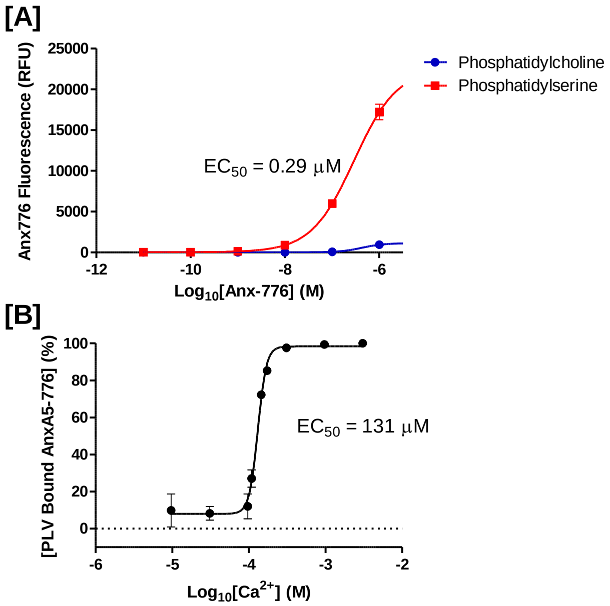

In vitro assays with coated phospholipid beads: **(A)** ANX776 has a greater affinity for phosphatidylserine (red) than phosphotidylcholine (blue) lipids in the presence of 2 mM calcium. **(B)** Binding of ANX776 to 400 µM $PC_{85\%}PS_{15\%}$ liposomes is calcium dependent.

Methods:

[A] Binding of ANX776 to Phosphatidylserine and phosphotidylcholine lipids

Phosphatidylserine (DPPS) or phosphatidylcholine (egg) were dissolved in ethanol (16.7 mg/mL) before 0.1 mg/well was added to a Nunc Maxisorb 96 well plate. Plates were left to evaporate overnight at room temperature leaving a lipid cake. Serial dilutions of ANX776 in HEPES saline buffer (10 mM HEPES, 140 mM Sodium Chloride, 2 mM Calcium chloride, pH 7.4) were added to wells and incubated at 37 °C for 30 minutes. Each well was washed gently three times with buffer solution before recording the plate fluorescence in a LI-COR Odyssey Near Infrared plate reader (800nm, 4.0 gain). Mean intensity of each well was recorded using FIJI ImageJ software and results (n=2) were fit to a four-parameter dose response curve with variable slope (R^2 = 0.998) from which EC_{50} was determined.

[B] Calcium dependence of ANX776 binding to Phosphatidylserine

13 mM liposomes were prepared as described previously(Davis *et al.*, 2014), PC85%PS15% (molar ratios) were dissolved in chloroform:methanol (5:1 ratio) before drying by rotary evaporation (45°C, 50 mBar, 1h). The resulting lipid film was rehydrated with HEPES saline buffer (10 mM HEPES, 150 mM Sodium chloride pH 7.4) at 45 °C for 1h. The resulting multilamellar solution was freeze-thawed 5 times in liquid nitrogen and extruded 10 times through polycarbonate filters 200 nm in diameter (Nucleopore Corp., Pleasanton, USA) using an extruder (Lipex Biomembranes Inc., Vancouver, Canada). Resulting liposomes were diluted to a working concentration of 400 µM in the presence of varying concentrations of calcium chloride (1 µM, 10 µM, 32 µM, 100 µM, 320 µM, 1 mM and 3.16 mM) or 0.1 mM EDTA (no calcium control). To each solution 800 nM of ANX776 was added, mixed by inversion before

centrifugation (100,000 g, 4°C 45 minutes) to pellet liposomes. Supernatant was discarded and liposomes disrupted by resuspension in the same volume of HEPES saline buffer with 1% Triton-X100. The amount of ANX776 in each assay was determined using a Li-core odyssey imaging system (800nm channel, gain 2.5) and intensities normalised using no ANX776 as 0% and 800 nM ANX776 as 100%. Results (n=3) were fit to a four-parameter dose response curve with variable slope (R^2 = 0.996) from which EC_{50} was determined.

Table S7: Medical Characteristics

Diagnostic and Medical Characteristics

	Glaucoma	Normal
Glaucoma Suspect	3 (62.5%)	0
Primary Open Angle Glaucoma	5 (12.5%)	0
Family history Glaucoma	1 (12.5%)	0
Glaucoma medication	5 (62.5%)	0
Comorbidities	4 (50%)[a]	3 (37.5%)[b]

a = Systemic Hypertension (4), Mild Depression, Coronary Heart Disease (2)

b = Systemic Hypertension (3), Hypercholesterolemia, Acquired Spondylolisthesis.

Table S8: Rates of Progression of Glaucoma Patients at Baseline and Final Follow-up

Subject (follow-up months)	Eye R/L	HRT Rim Area mm^2/year		RNFL 3.5 µm/year		RNFL 4.1 µm/year		RNFL 4.7 µm/year		MRW µm/year		MD dB/year		VFI %/year		Total
		Baseline	Final	Baseline	Final	Baseline	Final	Baseline	Final	Baseline	Final	Baseline	Final	Baseline	Final	
5 (13.5)	R			-2.8*	-2.1*											2
	L	-0.045*	-0.027**	-6.0**	-5.2**											4
6 (9.8)	R			-14.0*												1
	L	-0.02**			-5.5*											2
7 (13.0)	R		--0.018**									-2.87*	-2.08*			3
	L		-0.046**	-3.8*	-3.4**							-3.19*	-3.19*			5
8 (13.6)	R			-3.0*	-2.1*											2
	L			-6.1**	-4.0**	-5.6**	-3.7**	-5.6**	-4.2**	-7.0**	-5.5**				-5.127*	9
9 (11.9)	R					-4.4*				-23.2*						2
	L												-1.27*			1
11 (15.4)	L			-3.4*	-3.4**	-2.5*	-6.0**				-9.4*		-1.75*			6
13 (11.4)	R									-12.8**	-5.5*					2
	L				-2.2*											1
14 (15.1)	R			-4.0*	-2.5*	-3.3**		-2.5*	-1.9**							5
	L				-2.1**		-1.2*		-1.0*							3
Count		2	3	8	10	4	3	2	3	3	4	3	5	0	1	

Glaucoma progression was defined by a significant (*p<0.05; **p<0.01) negative slope in the rate of progression (RoP) of each parameter computed for HRT, OCT using retinal nerve fibre layer (RNFL) measurements at three different diameters from the optic disc (3.5, 4.1, and 4.7 mm), and Bruch's membrane opening minimum rim width and SAP MD and VFI. Patients were included in the study if at least one eye showed progression at baseline. Follow-up RoP are shown with red text highlighting an increase in the RoP compared to baseline. This table provides the raw data from which Table 1 and Figure 3B are derived.

Abbreviations: HRT (Heidelberg Retinal Tomography III); OCT (optical coherence tomography; Heidelberg Spectralis); SAP (standard automated perimetry; HFA 640i, Humphrey Field Analyzer); RNFL (retinal nerve fibre layer); MRW (Bruch's membrane opening minimum rim width); MD (mean deviation); VFI (visual field index)

Figure S9: DARC Fluorescent Images at in Healthy and Glaucoma Eyes

Retinal images from a glaucoma (A-D) patient and a healthy control (E-H) at baseline (A-B, E-F) and 240 minutes (C-D, G-H) following 0.4mg ANX776 injection, showing low (A, C, E, G) and high (B, D, F, H) magnification (scale bars indicated) retinal images. White spots are ANX-776 fluorescent-labelled retinal cells clearly seen with increased activity in glaucoma patient

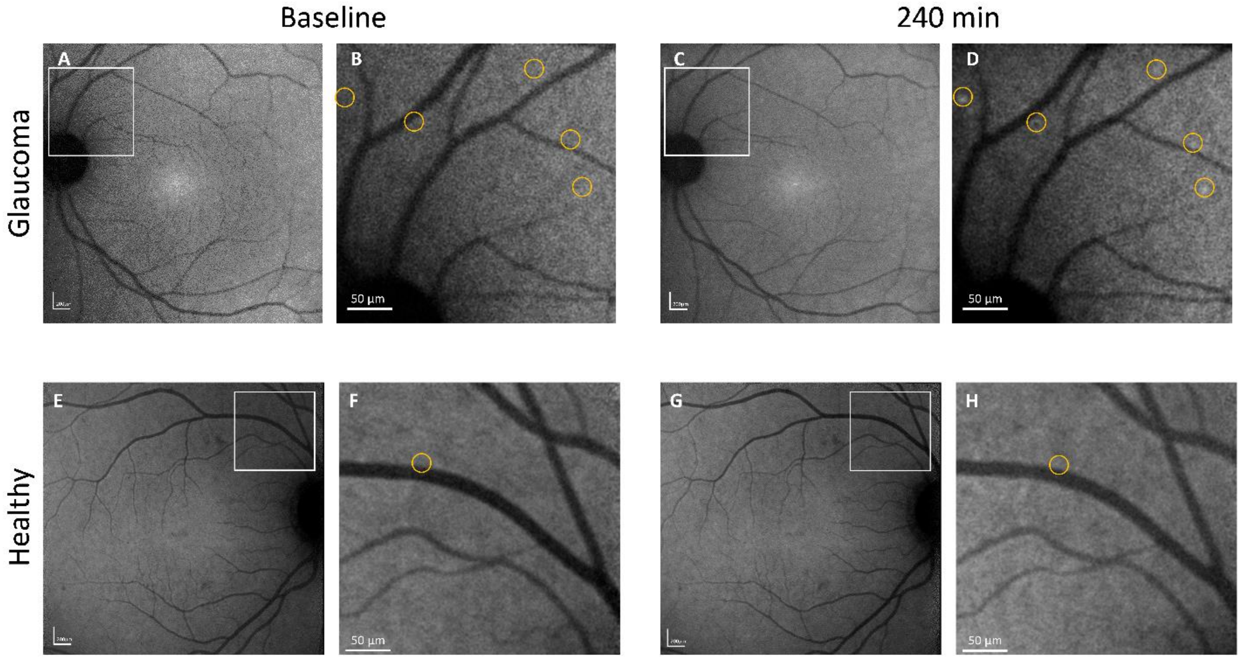

Figure S10: DARC Fluorescent Images at different ANX776 Doses in Glaucoma Eyes

(Note: reflective image in 0.2 mg baseline (RED frame) at same level as fluorescent and focusing on nerve fibres)

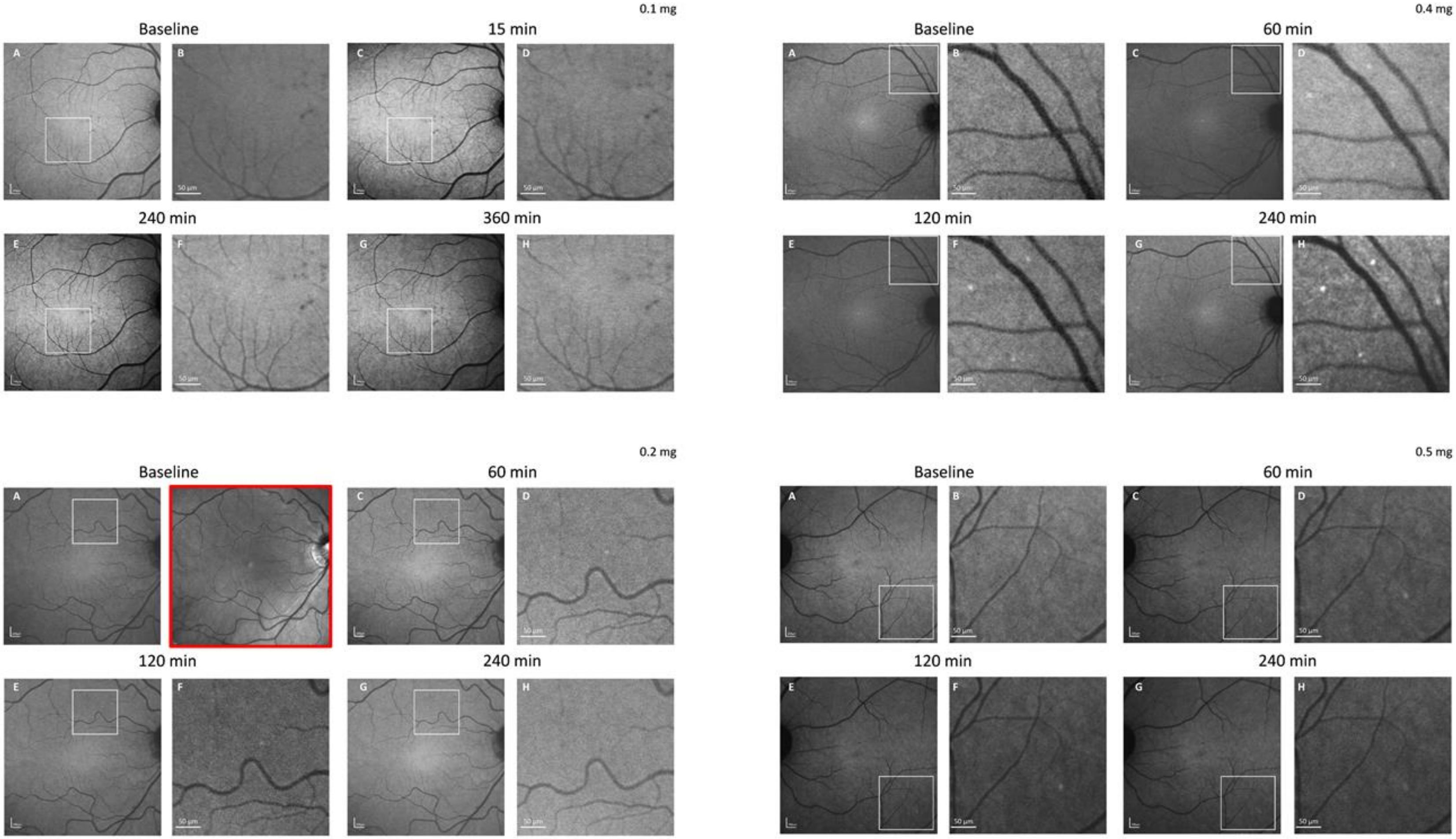

Figure S11: Development of Anx776 Positive Spots over Time

The bar charts below shows the analysis of the total number of unique Anx776 positive spots (labelled Total) and their first appearance (labelled New) at different time points at 15, 30, 60, 120, 240 and 360 minutes after ANX776 administration.

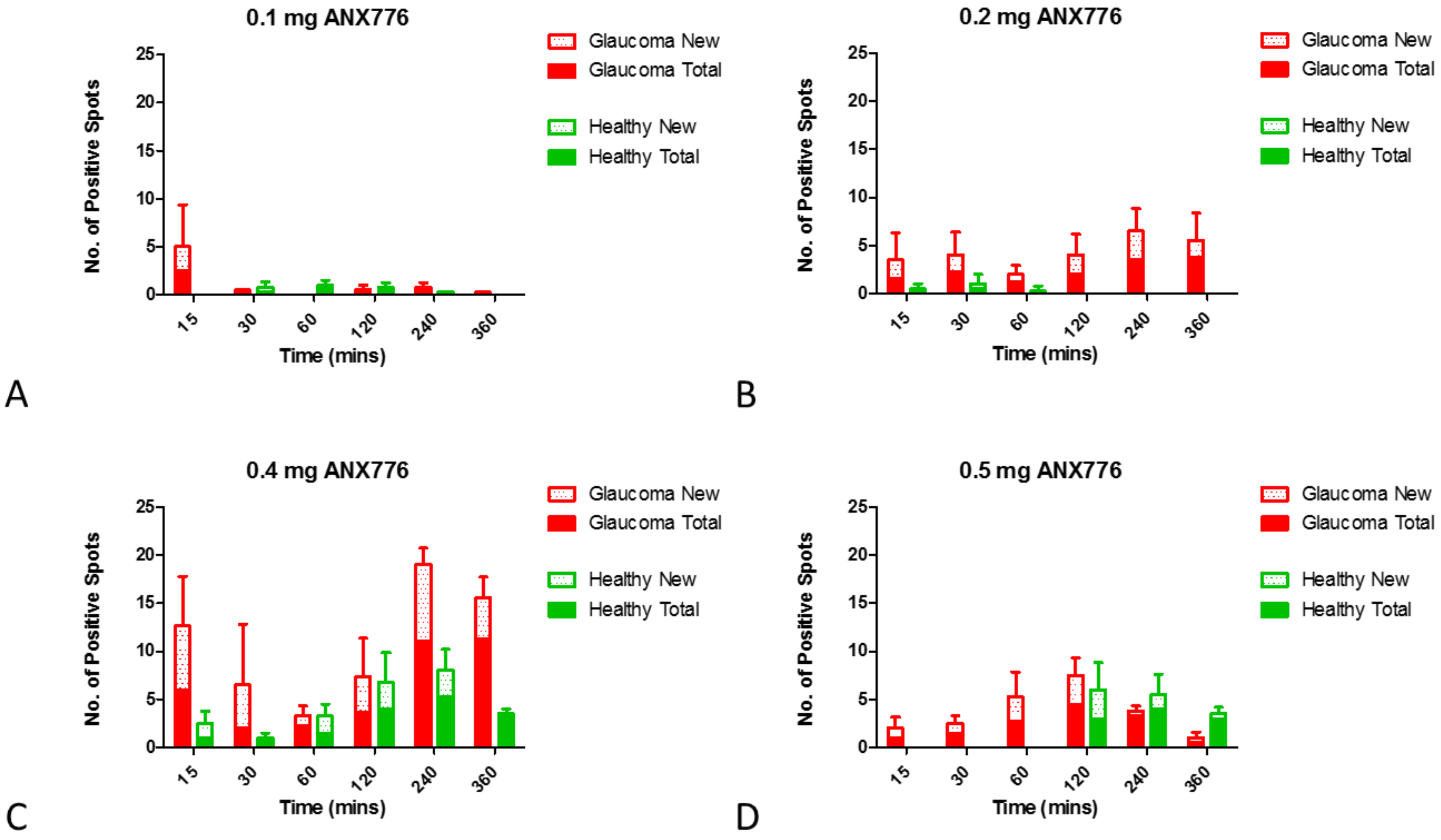

Table S12: Summary of Clinical Trials with Annexin 5

Reference	Objective and Design	Evaluation	Safety Data (AEs)
Hofstra et al., 2000(Hofstra *et al.*, 2000)	To study in-vivo cell death in the hearts of patients with an acute myocardial infarction using imaging with technetium-99m-labelled annexin-V-a protein that binds to cells undergoing apoptosis.	7 patients with an acute myocardial infarction and one control were treated by percutaneous transluminal coronary angioplasty, resulting in thrombolysis in myocardial infarction (TIMI) III flow of the infarct-related artery. 2 h after reperfusion, 1 mg annexin-V labelled with 584 MBq Tc-99m was injected intravenously. Early (mean 3.4 h) and late (mean 20.5 h) single-photon-emission computed tomographic (SPECT) images of the heart were obtained. Routine myocardial resting-perfusion imaging was also done to verify infarct localisation.	No observed safety issues arising from use of the product.
Narula et al., 2001(Narula *et al.*, 2001)	To look at cardiac allograft rejection	0.5 – 1.0 mg iv in 10 mg	The present study of 18 cardiac allograft recipients, 13 patients had negative and five had positive myocardial uptake of annexin. These latter five demonstrated at least moderate transplant rejection and caspase-3 staining, suggesting apoptosis in their biopsy specimens. This study reveals the clinical feasibility and safety of annexin-V imaging for noninvasive detection of transplant rejection by targeting cell membrane phospholipid alterations that are commonly associated with the process of apoptosis.
Hofstra et al., 2001(Hofstra *et al.*, 2001)	^{99m}TC-p-annexin-V was used to visualize apoptosis in patient with a cardiac tumour	1 patient, 1.0 mg iv	No adverse events identified in relation to the use of the annexin imaging agent, however, the patient in question who had part of the tumour removed, refused additional treatment and died 2.5 months later. Post mortem analysis indicating a large intracardiac tumour mass and multiple metastases.

Reference	Objective and Design	Evaluation	Safety Data (AEs)
Belhocine 2002(Belhocine *et al.*, 2002)	Fifteen patients presenting with lung cancer (n = 10), lymphoma (n = 3), or breast cancer (n = 2) underwent (99m)Tc-Annexin V scintigraphy before and within 3 days after their first course of chemotherapy.	Tumor response was evaluated by computed tomography and (18)F-fluoro-2-deoxy-D-glucose positron emission tomography scans, 3 months in average after completing the treatment. Median follow-up was 117 days. [1.0 mg iv in 2.5 ml]	No serious adverse events were observed.
Boersma 2003(Boersma *et al.*, 2003)	To evaluate the potential of (99m)Tc-AnxA5 for in vivo scintigraphy of apoptotic cells, the pharmacokinetics and imaging properties of two radiopharmaceuticals, (99m)Tc-(n-1-imino-4-mercaptobutyl)-AnxA5 (I-AnxA5) and (99m)Tc-(4,5-bis(thioacetamido)pentanoyl)-AnxA5 (B-AnxA5), were studied<br>Myocardial infarction, heart failure, other heart disease, healthy	12, 4, 3, 1 patients; 0.8 mg iv in 7 ml<br><br>AnxA5 was administered intravenously to seven patients and one healthy volunteer, and B-AnxA5 was administered to 12 patients. All patients in the pharmacokinetic study had myocardial disease. Additionally, imaging was performed in a patient with acute myocardial infarction, as well as in three patients with different malignancies.	The plasma concentration, excretion and biodistribution of (99m)Tc-AnxA5 were measured, as well as levels of AnxA5 antigen. The kinetic data of both radiopharmaceuticals in plasma fitted a two-compartment model. Both preparations had similar half-lives, but a different distribution over the two compartments. Plasma levels of AnxA5 antigen showed a broad variation. Both radiopharmaceuticals accumulated in the kidney, liver and gut. B-AnxA5 was excreted significantly faster than I-AnxA5. Both compounds can be used for imaging of the head/neck region, the thorax and the extremities. B-AnxA5 has a faster clearance and a lower radiation dose. Imaging of apoptosis in the abdomen will be difficult with both radiopharmaceuticals, and especially with B-AnxA5 because of its faster appearance in the gut.
Kemerink *et. al.* 2003(Kemerink *et al.*, 2003)	This study looked at safety and biodistribution of 99mTc-HYNIC-Annexin V, measuring renal and liver function, haematology, blood coagulation and vital signs, in order to calculate organ-absorbed doses and effective dose.	6 male healthy volunteers; 99mTc-Anx 25ug iv<br><br>Approximately 250 MBq of (99m)Tc-HYNIC-annexin V were injected intravenously, directly followed by a 30-min dynamic study. Whole-body scans were obtained at about 30 min, 3 h, 6 h, and 24 h after injection.	99mTc-HYNIC-AnxV was found to be a safe radiopharmaceutical in this study because none of the volunteers reported any adverse effect and no changes were observed in any of the safety parameters obtained before<br><br>and after administration

Reference	Objective and Design	Evaluation	Safety Data (AEs)
Van de Wiele 2003(van de Wiele *et al.*, 2003)	This study reports on the relationship between quantitative technetium-99m- (99mTc-) 6-hydrazinonicotinic (HYNIC) radiolabeled annexin V tumor uptake, and the number of tumor apoptotic cells derived from histologic analysis in patients suspected of primary or recurrent head and neck carcinoma	20 patients (18 male, 2 female)<br><br>All patients underwent a spiral computed tomography (CT) scan, 99mTc-HYNIC annexin V tomography, and subsequent surgical resection of the suspected primary or recurrent tumor.	No indication of any serious adverse events, however the publication does not provide any safety data or discussion.
Lahorte 2003(Lahorte *et al.*, 2003)	Investigation of the biodistribution and dosimetry for ^{123}I-rh-Anx in mice and healthy human subjects.	6 healthy subjects; ^{123}I-rh-Anx; 940 ug iv<br><br>6 healthy human subjects received ^{123}I-rh-Anx (345 MBq) and underwent whole body scans up to 21 hours post iv injection. Time activity curves were then generated for organs of interest	No indication of any serious safety concerns based on information in the abstract.
Thimister 2003(Thimister *et al.*, 2003)	The aim of this study was to correctly localize Annexin A5 uptake in vivo and to determine the area at risk in humans with acute Myocardial infarction.	9 patients; B-Anx c. 1 mg iv<br><br>Before reperfusion was achieved, (99m)Tc-sestamibi was injected intravenously. Myocardial (99m)Tc-sestamibi perfusion scintigraphy was performed after reperfusion. Thereafter, (99m)Tc-labeled annexin A5 was administered intravenously, followed by scintigraphic imaging of the heart. Myocardial (99m)Tc-sestamibi scintigraphy was repeated 1-3 wk after the MI onset. (99m)Tc-Annexin uptake was also studied in the subacute phase of the MI in 2 patients.	All subjects were patients with acute myocardial infarction. All patients recovered well after treatment in the coronary care unit. No side effects were observed after the injection of either $_{99m}$Tc-MIBI or $_{99m}$Tc-annexin A5.

Reference	Objective and Design	Evaluation	Safety Data (AEs)
Kartachova 2004(Kartachova *et al.*, 2004)	Identify visual patterns of uptake of 99mTc-HYNIC-rh-Annexin V by tumours to assess treatment response in patients with malignant lymphoma, leukaemia, NSCLC or H&NSCC.	33 patients underwent 99mTc-HYNIC-rh-Annexin V scintigraphy before and after radiotherapy, platinum-based chemotherapy or concurrent chemoradiation. Planar and SPECT images were visually examined to assess changes in tumour 99mTc-HYNIC-rh-Annexin V uptake. A 4-step scale was used to grade Annexin V uptake before and after treatment to assess tumour response after therapy.	No indication of any serious safety concerns based on information in the abstract.
Kietselaer 2004 (Kietselaer *et al.*, 2004)	Evaluation of detection of apoptosis of smooth muscle cells and macrophages in Athero-sclerotic plaques to identify instability in the plaque as a predictor of transient ischaemic attacks (TIAs).	4 patients with a recent or remote history of TIA were evaluated using 99mTc labelled Annexin V (600-800 MBq), receiving the product 1-3 days before carotid endarterectomy. 6 hours after infusion, images were obtained using SPECT	No indication of any serious adverse events, however the publication does not provide any safety data or discussion.

Reference	Objective and Design	Evaluation	Safety Data (AEs)
Vermeersch 2004a(Vermeersch *et al.*, 2004a)	The aim of this study was to estimate the intra-, inter-, and day-to-day reproducibility of quantitative 99mTc-HYNIC annexin-V tumor uptake values in patients suffering from head and neck carcinomas.	13 patients suffering from clinically suspected histologically confirmed squamous head and neck carcinomas were prospectively included in the study. All patients were scheduled to undergo a spiral computed tomography scan and two 99mTc-HYNIC annexin-V scintigraphies within 3-5 days from each other, referred to as day 1 and day 2 of scintigraphy. The percentage of uptake of the injected dose of 99mTc-HYNIC annexin-V in tumor lesions on scintigrams divided by the tumor volume, as derived from CT, was determined twice within an interval of 2 weeks by observer 1 and once by observer 2 on day 1 of scintigraphy and once on day 2 of scintigraphy by observer 1.	No indication of any serious adverse events, however the publication does not provide any safety data or discussion.
Vermeersch 2004b(Vermeersch *et al.*, 2004b)	Head and neck cancers<br>The potential of 99mTc-HYNIC Annexin-V scintigraphy to visualize primary head and neck carcinoma was assessed and compared with computed tomography (CT) findings and histology	18 patients; HYNIC Anx 0.25 mg<br>Eighteen patients suspected of having primary head and neck carcinoma underwent a spiral CT scan and 99mTc-HYNIC Annexin-V scintigraphy within 1 week of each other, followed by resection of the suspected lesion. Results obtained by CT and scintigraphy were compared vs. histopathology. The diagnosis was primary head and neck carcinoma in 18 patients, accompanied by lymph node involvement in seven patients.	No indication of any serious adverse events, however the publication does not provide any safety data or discussion.

Reference	Objective and Design	Evaluation	Safety Data (AEs)
Vermeersch 2004c(Vermeersch *et al.*, 2004c)	Head and neck cancers<br>This study reports on the relationship between quantitative (99m)Tc-HYNIC radiolabelled annexin V tumour uptake measurements, Fas ligand (FasL) expression, matrix metalloproteinase-9 (MMP-9) expression, microvessel density (MVD) and the number of tumour-infiltrating lymphocytes in squamous cell carcinoma of the head and neck (SCCHN) patients.	28 patients; HYNIC Anx 0.25 mg<br>Twenty-eight patients (24 men and 4 women; mean age 59 years, range 43-83 years) suffering from a primary (n, number of patients=22) or locally recurrent (n=6) SCCHN were studied. All patients underwent a spiral CT scan, allowing estimation of lesion size in three dimensions, and (99m)Tc-HYNIC annexin V scintigraphy within 1 week of each other. Biopsies or resection of the suspected primary tumour or local recurrence for histopathological analysis were performed on all patients within a period of 10 days following (99m)Tc-HYNIC annexin V scintigraphy.	No indication of any serious adverse events, however the publication does not provide any safety data or discussion.
Haas 2004(Haas *et al.*, 2004)	To evaluate (99m)Tc-Annexin-V (TAV) scintigraphy in monitoring radiation-induced apoptotic cell death in follicular lymphoma (FL) patients.	11 follicular lymphoma patients (female, 4 male) underwent TAV imaging before and 24h after radiotherapy The TAV scintigraphy (total body studies and SPECT of the irradiated sites) was performed 4 hours after the administration of the radiopharmaceutical. Tumor uptake was scored in a semiquantitative manner as absent, weak, present, or intense with corresponding categories for the cytologic slides. Response evaluation was performed after 1 week and 4 weeks both in terms of completeness and speed of remission.	No indication of any serious safety concerns based on information in the abstract.

Reference	Objective and Design	Evaluation	Safety Data (AEs)
Rongen 2005 (Rongen *et al.*, 2005)	The aim of the study was to set up an easy-to-use human in vivo model to study ischemic or pharmacological preconditioning.	44 healthy male subjects performed unilateral ischemic handgrip.; At reperfusion, technetium-99m-labeled Annexin A5 (0.1mg) was injected intravenously as a marker of ischemic injury, and both forearms and hands imaged simultaneously with a gamma camera to analyse regions of interest	Retention of radiolabeled albumin in the experimental arm was not observed, indicating that the observed targeting of Annexin A5 is not caused by nonspecific targeting such as changes in vascular permeability or reactive hyperemia. Delay of the Annexin A5 injection by 1 hour did not result in uptake of Annexin A5. In a pilot study in 6 healthy volunteers, the AV difference in plasma creatine kinase activity across the experimental forearm did not change in response to this ischemic exercise, excluding relevant skeletal muscle necrosis.<br><br>No indication of any serious safety concerns based on information in the publication.
Lampl 2006 (Lampl *et al.*, 2006)	To use radiolabelled Annexin V to image Alzheimer dementia (AD)	12 patients (5 with AD, 7 with non-AD) received HYNIC Anx 0.1 mg. There were 6 control patients.	No indication of any serious safety concerns based on information in the abstract.
Lorberboym 2006 (Lorberboym *et al.*, 2006)	To determine the ability of radiolabeled annexin V to concentrate at sites of ischemic injury in patients with acute cerebral stroke and to correlate annexin V imaging in these patients with the degree of blood-brain barrier (BBB) breakdown.	12 patients with acute stroke had a complete neurological examination including non-contrast CT scan. The patients then received annexin V and a SPECT of the brain obtained 2hrs after injection. The integrity of the BBB was evaluated in 7 patients using Tc-99m-DTPA brain SPECT.	No indication of any serious safety concerns based on information in the abstract.

Reference	Objective and Design	Evaluation	Safety Data (AEs)
Kartachova 2006 (Kartachova *et al.*, 2006)	To map treatment-induced (99m)Tc-Hynic-rh-annexin V uptake in normal tissues using co-registration of SPECT and CT.	19 patients (11 male, 8 female) with various malignant tumours (12 lymphomas, 4 NSCLC and 3 H&NSCC) underwent (99m)Tc-Hynic-rh-annexin V scintigraphy and CT before and within 48 h after the start of anticancer therapy. SPECT and CT were performed separately, with the patient in a reproducible position. Volume-based automated and manual methods were used to match functional and anatomical data. SPECT/CT co-registration was used to evaluate treatment-induced changes in the normal structures.	A significant radiation field-related increase in early post-treatment (99m)Tc-Hynic-rh-annexin V uptake in salivary glands and bone marrow was detected in eight of nine patients. Radiation field-related increase in bone marrow activity above the baseline value was detected in all 13 irradiated patients. A minimal, symmetrical increase in activity in the salivary glands was detected after the initial course of platinum-based chemotherapy, and a diffuse prominent increase in (99m)Tc-Hynic-rh-annexin V in the bone marrow was detected in all cases. Precise delineation between the tumour and normal tissue tracer accumulation was accomplished in all cases using SPECT/CT co-registered volumes, enhanced by the "colourwash" technique.<br><br>No indication of any serious adverse events, however the publication does not provide any safety data or discussion.
Kartachova 2007 (Kartachova *et al.*, 2007)	To evaluate if sequential 99mTc Hynic-rh- annexin V scintigraphy (TAS) can predict outcome in patients with advanced lung cancer, shortly after the start of platinum-based chemotherapy.	In 16 consecutive chemotherapy-naive patients with advanced stage non-small-cell lung cancer scheduled for platinum-based chemotherapy, TAS was performed before and within 48 hours after the start of therapy. Chemotherapy-induced changes in tumor annexin V uptake were compared with treatment response determined according to Response Evaluation Criteria in Solid Tumors.	A significant correlation ($r2 = 0.86$; $P = .0001$) was found between annexin V metabolic changes and treatment outcome. All patients with notably increased annexin V tumour uptake showed complete or partial response. Less prominently increased or decreased uptake correlated with stable or progressive disease.<br><br>No indication of any serious adverse events, however the publication does not provide any safety data or discussion.
Kietselaer 2007 (Kietselaer *et al.*, 2007)	To evaluate the role of annexin A5 imaging for detection of apoptosis (programmed cell death) in heart failure patients	Annexin A5 imaging was performed on 9 consecutive heart failure patients with advanced nonischemic cardiomyopathy (dilated, n = 8; hypertrophic, n = 1) and in 2 relatives having the same genetic background as the hypertrophic cardiomyopathy patient but no heart failure.	No indication of any serious adverse events, however the publication does not provide any safety data or discussion.

Reference	Objective and Design	Evaluation	Safety Data (AEs)
Van den Brande 2007 (Van den Brande *et al.*, 2007)	To test whether the ability of rapid anti-TNF-induced apoptosis in the gut predicts the efficacy of anti-TNF treatment in inflammatory bowel disease.	(99m)Technetium-annexin V single-photon emission computer tomography (SPECT) was performed in 2 models of murine experimental colitis and in 14 patients with active Crohn's disease to study the effect of anti-TNF treatment on apoptosis in the intestine during active colitis. Disease activity was evaluated 2 weeks after infliximab infusion using the CDAI (definition response: drop of >100 points).	No indication of any serious adverse events, however the publication does not provide any safety data or discussion.<br><br>It was investigated whether the accumulation of 99mTc–annexin V was different in patients exhibiting a favourable response to infliximab from those who did not display an objective response. The CUR increased in both responders and non-responders, but interestingly the increase in CUR was markedly higher in the group of responders. In 10 responding patients, the mean increase in CUR was 98.6%, compared with 15.2% in the 4 non-responding patients ($p=0.03$)
Kartachova 2008 (Kartachova *et al.*, 2008)	To determine the reliability of visual analysis of 99mTc-HYNIC-rh-annexin-V tumour uptake (ATU) compared to quantitative tracer uptake evaluation.	38 patients (22 male, 16 female) with histologically proved lymphoma (n=31), NSCLC (n=4) and H&NSCC (n=3) were examined. 99mTc-HYNIC-rh-annexin-V scintigraphy (TAS) was acquired before and within 2 days after the start of anti-cancer treatment. Maximal counts per pixel in the tumour volume (Cmax) were calculated for every target lesion. To match the quantitative and visual ATU, both were expressed as a four-grade score.	No indication of any serious adverse events, however the publication does not provide any safety data or discussion.
Kurihara 2008 (Hashizume *et al.*, 2008)	To use (99m)Tc EC-annexin V to image tumor cells undergoing apoptosis.	In 10 patients with breast cancer, scintigraphic images and dosimetric estimates were obtained after administering 99mTc-ethylenedicysteine-Annexin V	Nine of the 10 cases showed detectable (99m)Tc EC-annexin V uptake in tumor. Higher values of T/N ratios are associated with patient after treatment. This indicates that apoptosis can be quantified using (99m)Tc EC-annexin V.

Reference	Objective and Design	Evaluation	Safety Data (AEs)
Hoebers 2008 (Hoebers *et al.*, 2008)	To determine the value of (99m)Tc Hynic-rh-Annexin-V-Scintigraphy (TAVS), a non-invasive in vivo technique to demonstrate apoptosis in patients with head and neck squamous cell carcinoma.	TAVS were performed before and within 48 h after the first course of cisplatin-based chemoradiation. Single-photon emission tomography data were co-registered to planning CT scan. Complete sets of these data were available for 13 patients. The radiation dose at post-treatment TAVS was calculated for several regions of interest (ROI): primary tumour, involved lymph nodes and salivary glands. Annexin uptake was determined in each ROI, and the difference between post-treatment and baseline TAVS represented the absolute Annexin uptake: Delta uptake (DeltaU).	In 24 of 26 parotid glands, treatment-induced Annexin uptake was observed. Mean DeltaU was significantly correlated with the mean radiation dose given to the parotid glands (r = 0.59, p = 0.002): Glands that received higher doses showed more Annexin uptake. DeltaU in primary tumour and pathological lymph nodes showed large inter-patient differences. A high correlation was observed on an inter-patient level (r = 0.71, p = 0.006) between the maximum DeltaU in primary tumour and in the lymph nodes.<br><br>Within the dose range of 0-8 Gy, Annexin-V-scintigraphy showed a radiation-dose-dependent uptake in parotid glands, indicative of early apoptosis during treatment. The inter-individual spread in Annexin uptake in primary tumours could not be related to differences in dose or tumour volume, but the Annexin uptake in tumour and lymph nodes were closely correlated. This effect might represent a tumour-specific apoptotic response.

Table S13: Supplementary Statistical details

<u>Summary of potential models for total unique DARC count</u>

Six possible models are summarised, the first two models apply to the results quoted in the main text, 'ln' here refers to natural log.

1) $\ln(Y_{ijkl}) = \mu + g_i + d_j + e_{ijkl}$
Y_{ijkl} = lth eye measurement of response from patient k who received dose j and had glaucoma status i.

μ = constant
i = glaucoma subscript, i=1,2 status 0,1 (healthy, glaucoma)
j=dose subscript, j=1,2,3,4, dose - 0.1, 0.2, 0.4, 0.5mg
k = patient subscript, k=1-15
l = eye subscript, l=1,2 right and left eyes
e_{ijkl} random error per eye

2) $\ln(Y_{ijkl}) = \mu + g_i + \ln(d_j) + e_{ijkl}$
as 1) but with dose fitted as a continuous covariate

3) $\ln(Y_{ijkl}) = \mu + g_i + d_j + p_k + e_{ijkl}$
$p_k \sim N(0, \sigma_k)$ included as a random patient effect

4) $\ln(Y_{ijkl}) = \mu + g_i + \ln(d_j) + p_k + e_{ijkl}$
as 3) but with dose fitted as a continuous covariate

5) $\ln(Z_{ijk}) = \mu + g_i + d_j + e_{ijk}$
Z_{ijk} = maximum count of 2 eyes, excluding patient with only 1 eye measurement, k=1-14

6) $\ln(Z_{ijk}) = \mu + g_i + \ln(d_j) + e_{ijk}$
as 5) but with dose fitted as a continuous covariate

Model	Glaucoma effect	P-value	Dose effect P-value (dof)
1	2.35	0.008	P<0.001 (3)
2	2.07	0.036	P<0.001 (1)
3	2.26	0.033	P<0.001 (3)
4	2.06	0.089	P<0.001 (1)
5	2.92	0.046	P=0.002 (3)
6	2.46	0.082	P=0.005 (1)

The glaucoma effect is expressed as the fold difference, $\exp(g_2)$.
dof = degrees of freedom

Video S14: Supplementary Procedural Video

The DARC procedure consists of the acquisition of retinal images recorded at baseline and at 15 to 360 minutes after intravenous administration of ANX776. DARC spots on the retinal images are seen as “white” hyperfluorescent spots on the retina with maximal numbers seen at 240-360 minutes after intravenous administration of ANX776

Link to video:

https://www.dropbox.com/s/08jba2h5h6wiv43/Darc%20video%20short%20version.mov?dl=0

References

Belhocine T, Steinmetz N, Hustinx R, Bartsch P, Jerusalem G, Seidel L*, et al.* Increased uptake of the apoptosis-imaging agent (99m)Tc recombinant human Annexin V in human tumors after one course of chemotherapy as a predictor of tumor response and patient prognosis. Clin Cancer Res 2002; 8(9): 2766-74.

Boersma HH, Liem IH, Kemerink GJ, Thimister PW, Hofstra L, Stolk LM*, et al.* Comparison between human pharmacokinetics and imaging properties of two conjugation methods for 99mTc-annexin A5. Br J Radiol 2003; 76(908): 553-60.

Borrie SC, Cheung W, Guo L, Barber AJ, Singh RSJ, Gardner TW*, et al.* Diabetic retinal neurodegeneration: In vivo imaging of retinal ganglion cell apoptosis in the Ins2akita/j mouse. Investigative Ophthalmology and Visual Science 2008; 49: ARVO E-Abstract 4924.

Cordeiro MF, Guo L, Coxon KM, Duggan J, Nizari S, Normando EM*, et al.* Imaging multiple phases of neurodegeneration: a novel approach to assessing cell death in vivo. Cell Death Dis 2010; 1: e3.

Cordeiro MF, Guo L, Luong V, Harding G, Wang W, Jones HE*, et al.* Real-time imaging of single nerve cell apoptosis in retinal neurodegeneration. Proceedings of the National Academy of Sciences of the United States of America 2004; 101(36): 13352-6.

Davis BM, Normando EM, Guo L, Turner LA, Nizari S, O'Shea P*, et al.* Topical delivery of Avastin to the posterior segment of the eye in vivo using annexin A5-associated liposomes. Small 2014; 10(8): 1575-84.

Galvao J, Davis B, Tilley M, Normando E, Duchen MR, Cordeiro MF. Unexpected low-dose toxicity of the universal solvent DMSO. FASEB J 2014; 28(3): 1317-30.

Galvao J, Elvas F, Martins T, Cordeiro MF, Ambrosio AF, Santiago AR. Adenosine A3 receptor activation is neuroprotective against retinal neurodegeneration. Exp Eye Res 2015; 140: 65-74.

Guo L, Davis B, Nizari S, Normando EM, Shi H, Galvao J*, et al.* Direct optic nerve sheath (DONS) application of Schwann cells prolongs retinal ganglion cell survival in vivo. Cell Death Dis 2014; 5: e1460.

Guo L, Moss SE, Alexander RA, Ali RR, Fitzke FW, Cordeiro MF. Retinal ganglion cell apoptosis in glaucoma is related to intraocular pressure and IOP-induced effects on extracellular matrix. Invest Ophthalmol Vis Sci 2005; 46(1): 175-82.

Guo L, Salt TE, Luong V, Wood N, Cheung W, Maass A*, et al.* Targeting amyloid-beta in glaucoma treatment. Proceedings of the National Academy of Sciences of the United States of America 2007; 104(33): 13444-9.

Guo L, Salt TE, Maass A, Luong V, Moss SE, Fitzke FW*, et al.* Assessment of neuroprotective effects of glutamate modulation on glaucoma-related retinal ganglion cell apoptosis in vivo. Invest Ophthalmol Vis Sci 2006; 47(2): 626-33.

Haas RL, de Jong D, Valdes Olmos RA, Hoefnagel CA, van den Heuvel I, Zerp SF*, et al.* In vivo imaging of radiation-induced apoptosis in follicular lymphoma patients. Int J Radiat Oncol Biol Phys 2004; 59(3): 782-7.

Hashizume K, Hirasawa M, Imamura Y, Noda S, Shimizu T, Shinoda K*, et al.* Retinal dysfunction and progressive retinal cell death in SOD1-deficient mice. Am J Pathol 2008; 172(5): 1325-31.

Hoebers FJ, Kartachova M, de Bois J, van den Brekel MW, van Tinteren H, van Herk M*, et al.* 99mTc Hynic-rh-Annexin V scintigraphy for in vivo imaging of apoptosis in patients with head and neck cancer treated with chemoradiotherapy. Eur J Nucl Med Mol Imaging 2008; 35(3): 509-18.

Hofstra L, Dumont EA, Thimister PW, Heidendal GA, DeBruine AP, Elenbaas TW*, et al.* In vivo detection of apoptosis in an intracardiac tumor. Jama 2001; 285(14): 1841-2.

Hofstra L, Liem IH, Dumont EA, Boersma HH, van Heerde WL, Doevendans PA*, et al.* Visualisation of cell death in vivo in patients with acute myocardial infarction. Lancet 2000; 356(9225): 209-12.

Kartachova M, Haas RL, Olmos RA, Hoebers FJ, van Zandwijk N, Verheij M. In vivo imaging of apoptosis by 99mTc-Annexin V scintigraphy: visual analysis in relation to treatment response. Radiother Oncol 2004; 72(3): 333-9.

Kartachova M, van Zandwijk N, Burgers S, van Tinteren H, Verheij M, Valdes Olmos RA. Prognostic significance of 99mTc Hynic-rh-annexin V scintigraphy during platinum-based chemotherapy in advanced lung cancer. J Clin Oncol 2007; 25(18): 2534-9.

Kartachova MS, Valdes Olmos RA, Haas RL, Hoebers FJ, van den Brekel MW, van Zandwijk N*, et al.* Mapping of treatment-induced apoptosis in normal structures: 99mTc-Hynic-rh-annexin V SPECT and CT image fusion. Eur J Nucl Med Mol Imaging 2006; 33(8): 893-9.

Kartachova MS, Valdes Olmos RA, Haas RL, Hoebers FJ, van Herk M, Verheij M. 99mTc-HYNIC-rh-annexin-V scintigraphy: visual and quantitative evaluation of early treatment-induced apoptosis to predict treatment outcome. Nuclear medicine communications 2008; 29(1): 39-44.

Kemerink GJ, Liu X, Kieffer D, Ceyssens S, Mortelmans L, Verbruggen AM*, et al.* Safety, biodistribution, and dosimetry of 99mTc-HYNIC-annexin V, a novel human recombinant annexin V for human application. J Nucl Med 2003; 44(6): 947-52.

Kietselaer BL, Reutelingsperger CP, Boersma HH, Heidendal GA, Liem IH, Crijns HJ*, et al.* Noninvasive detection of programmed cell loss with 99mTc-labeled annexin A5 in heart failure. J Nucl Med 2007; 48(4): 562-7.

Kietselaer BL, Reutelingsperger CP, Heidendal GA, Daemen MJ, Mess WH, Hofstra L*, et al.* Noninvasive detection of plaque instability with use of radiolabeled annexin A5 in patients with carotid-artery atherosclerosis. N Engl J Med 2004; 350(14): 1472-3.

Lahorte CM, van de Wiele C, Bacher K, van den Bossche B, Thierens H, van Belle S*, et al.* Biodistribution and dosimetry study of 123I-rh-annexin V in mice and humans. Nuclear medicine communications 2003; 24(8): 871-80.

Lampl Y, Lorberboym M, Blankenberg FG, Sadeh M, Gilad R. Annexin V SPECT imaging of phosphatidylserine expression in patients with dementia. Neurology 2006; 66(8): 1253-4.

Lorberboym M, Blankenberg FG, Sadeh M, Lampl Y. In vivo imaging of apoptosis in patients with acute stroke: correlation with blood-brain barrier permeability. Brain Res 2006; 1103(1): 13-9.

Maass A, von Leithner PL, Luong V, Guo L, Salt TE, Fitzke FW*, et al.* Assessment of Rat and Mouse RGC Apoptosis Imaging in Vivo with Different Scanning Laser Ophthalmoscopes. Curr Eye Res 2007; 32(10): 851-61.

Narula J, Acio ER, Narula N, Samuels LE, Fyfe B, Wood D*, et al.* Annexin-V imaging for noninvasive detection of cardiac allograft rejection. Nat Med 2001; 7(12): 1347-52.

Normando EM, Davis BM, De Groef L, Nizari S, Turner LA, Ravindran N*, et al.* The retina as an early biomarker of neurodegeneration in a rotenone-induced model of Parkinson's disease: evidence for a neuroprotective effect of rosiglitazone in the eye and brain. Acta Neuropathologica Communications 2016; in press.

Normando EM, Turner LA, Cordeiro MF. Imaging in Dry AMD. Drug Discovery Today: Therapeutic Strategies 2013; 10(1): e35 - e41.

Rongen GA, Oyen WJ, Ramakers BP, Riksen NP, Boerman OC, Steinmetz N*, et al.* Annexin A5 scintigraphy of forearm as a novel in vivo model of skeletal muscle preconditioning in humans. Circulation 2005; 111(2): 173-8.

Salt TE, Nizari S, Cordeiro MF, Russ H, Danysz W. Effect of the Abeta aggregation modulator MRZ-99030 on retinal damage in an animal model of glaucoma. Neurotox Res 2014; 26(4): 440-6.

Schmitz-Valckenberg S, Guo L, Cheung W, Moss SE, Fitzke FW, Cordeiro MF. [In vivo imaging of retinal cell apoptosis following acute light exposure.]. Ophthalmologe 2009.

Schmitz-Valckenberg S, Guo L, Maass A, Cheung W, Vugler A, Moss SE*, et al.* Real-time in vivo imaging of retinal cell apoptosis after laser exposure. Invest Ophthalmol Vis Sci 2008; 49(6): 2773-80.

Thimister PW, Hofstra L, Liem IH, Boersma HH, Kemerink G, Reutelingsperger CP*, et al.* In vivo detection of cell death in the area at risk in acute myocardial infarction. J Nucl Med 2003; 44(3): 391-6.

van de Wiele C, Lahorte C, Vermeersch H, Loose D, Mervillie K, Steinmetz ND*, et al.* Quantitative tumor apoptosis imaging using technetium-99m-HYNIC annexin V single photon emission computed tomography. J Clin Oncol 2003; 21(18): 3483-7.

Van den Brande JM, Koehler TC, Zelinkova Z, Bennink RJ, te Velde AA, ten Cate FJ*, et al.* Prediction of antitumour necrosis factor clinical efficacy by real-time visualisation of apoptosis in patients with Crohn's disease. Gut 2007; 56(4): 509-17.

Vermeersch H, Ham H, Rottey S, Lahorte C, Corsetti F, Dierckx R*, et al.* Intraobserver, interobserver, and day-to-day reproducibility of quantitative 99mTc-HYNIC annexin-V imaging in head and neck carcinoma. Cancer biotherapy & radiopharmaceuticals 2004a; 19(2): 205-10.

Vermeersch H, Loose D, Lahorte C, Mervillie K, Dierckx R, Steinmetz N*, et al.* 99mTc-HYNIC Annexin-V imaging of primary head and neck carcinoma. Nuclear medicine communications 2004b; 25(3): 259-63.

Vermeersch H, Mervillie K, Lahorte C, Loose D, Dierck RA, Steinmetz N*, et al.* Relationship of 99mTc-HYNIC annexin V uptake to microvessel density, FasL and MMP-9 expression, and the number of tumour-infiltrating lymphocytes in head and neck carcinoma. Eur J Nucl Med Mol Imaging 2004c; 31(7): 1016-21.
