# Supplementary material for: Real-time imaging of single neuronal cell apoptosis in patients with glaucoma
Source: Brain. 2017 Apr 26;140(6):1757–67. doi: 10.1093/brain/awx088 (PMC5445254; doi:10.1093/brain/awx088)
Supplement: Supplementary Data [file awx088_Supp.zip › awx088-suppl_data/brain-2016-02139-File007.pdf]

Video 1: ANX776 positive labelled cells are visualised *in vivo* with time-lapse video showing the development of DARC spots over time

Video with unmarked (left) and marked (right) panels showing the appearance of ANX776 positive “white” hyperfluorescent spots on the retina over time, with yellow rings highlighting spots on right panel and timeline as indicated. DARC spots appear throughout with maximal numbers at 240-360 minutes after intravenous administration of ANX776.
